# Borders of *Cis*-Regulatory DNA Sequences Preferentially Harbor the Divergent Transcription Factor Binding Motifs in the Human Genome

**DOI:** 10.3389/fgene.2018.00571

**Published:** 2018-11-22

**Authors:** Jia-Hsin Huang, Ryan Shun-Yuen Kwan, Zing Tsung-Yeh Tsai, Tzu-Chieh Lin, Huai-Kuang Tsai

**Affiliations:** ^1^Institute of Information Science, Academia Sinica, Nankang, Taipei, Taiwan; ^2^Department of Computational Medicine and Bioinformatics, University of Michigan, Ann Arbor, MI, United States

**Keywords:** transcription factor binding sites, motifs, *cis*-regulatory elements, TF binding specificities, open chromatins

## Abstract

Changes in *cis*-regulatory DNA sequences and transcription factor (TF) repertoires provide major sources of phenotypic diversity that shape the evolution of gene regulation in eukaryotes. The DNA-binding specificities of TFs may be diversified or produce new variants in different eukaryotic species. However, it is currently unclear how various levels of divergence in TF DNA-binding specificities or motifs became introduced into the *cis*-regulatory DNA regions of the genome over evolutionary time. Here, we first estimated the evolutionary divergence levels of TF binding motifs and quantified their occurrence at DNase I-hypersensitive sites. Results from our *in silico* motif scan and experimentally derived chromatin immunoprecipitation (TF-ChIP) show that the divergent motifs tend to be introduced in the edges of *cis*-regulatory regions, which is probably accompanied by the expansion of the accessible core of promoter-associated regulatory elements during evolution. We also find that the genes neighboring the expanded *cis*-regulatory regions with the most divergent motifs are associated with functions like development and morphogenesis. Accordingly, we propose that the accumulation of divergent motifs in the edges of *cis*-regulatory regions provides a functional mechanism for the evolution of divergent regulatory circuits.

## Introduction

Transcription factors (TFs) are primary regulators of gene expression that function by interacting with DNA in a sequence-specific manner. The capacity of a TF to recognize particular patterns of nucleotides (i.e., motifs) via DNA-binding domains is defined as the TF’s DNA-binding specificity (Jolma et al., 2013). Previous studies have reported that the DNA-binding specificities of TF orthologs between human and *Drosophila* are mostly conserved (Nitta et al., 2015). Nonetheless, TFs do evolve divergent binding specificities in different species through genetic variation, such as gene duplication and the expansion of gene families (Jolma and Taipale, 2011; Weirauch et al., 2014; Nitta et al., 2015). Divergence in TF binding specificities contributes significantly to differential gene regulation, and shapes eukaryotic evolution (Wittkopp and Kalay, 2012; De Mendoza et al., 2013; Schmitz et al., 2016).

In eukaryotic cells, multiple TFs interact cooperatively with genomic DNA to temporally and spatially regulate gene expression. Most eukaryotic chromatin is packed into nucleosomes, whereas active *cis*-regulatory elements have functional TF binding sites in nucleosome-depleted regions, where DNA is hypersensitive to cleavage by DNase I. DNase I hypersensitive sites (DHSs) have been studied extensively and are found to overlap with most TF binding sites (TFBSs) in a wide range of organisms. Major advances in the ENCODE project have used DHSs to map active *cis*-regulatory elements in the human genome (The Encode Project Consortium, 2012; Thurman et al., 2012). Integrative analyses using ENCODE data have identified hundreds of TF binding motifs (Wang et al., 2013; Yan et al., 2013) and extended the repertoire of TFs in the human genome (Lambert et al., 2018). However, there is high turnover in *cis*-regulatory sequences (Weirauch and Hughes, 2011) and over longer timescales, rapid and flexible transcription factor binding site (TFBS) gain and loss events occur between closely related species (Dowell, 2010; Shibata et al., 2012; Villar et al., 2014).

From a functional genomics perspective, the interplay between TF binding events and *cis*-regulatory regions is a pivotal step that allows transcriptional regulation to be rewired through evolutionary time. Many general properties of regulatory genomes rely on the broad presence of clustered TFBSs in *cis*-regulatory regions (Wang et al., 2012; Chen et al., 2015). The divergence of *cis*-regulatory sequences harboring various TFBSs and alterations of TF DNA-binding specificities have been proposed as the major driving forces of phenotypic change (Zheng et al., 2011; Deplancke et al., 2016; Schmitz et al., 2016). However, the manner by which DNA sequence changes in *cis*-regulatory regions could arise as a result of harboring diversified TF binding motifs remains unclear. Since a given region of DNA sequences can harbor more than one TF binding motif, the evolvability within *cis*-regulatory DNA sequences of a range of TF binding motifs has not been systematically studied.

To address this knowledge gap, we have developed a novel measurement, the motif prevalence index (MPI), for the level of divergence of motifs among eukaryotes, based on the discovery that TF binding motifs are generally conserved among diverse organisms. The method integrates the phylogenetic relationship between TF orthologs among animals and a comprehensive collection of TF binding motifs to compute the prevalence of human motifs across metazoan evolution using the Cis-BP database (Weirauch et al., 2014), which provides stringent inferences for TF binding motifs in diverse organisms. By averaging the MPI of all the motifs in the DNA region, we can study the evolution of DNA sequence preference in a range of TF DNA-binding motifs. Our results showed that the preference of the divergent motifs tends to locate in the borders of the open-chromatin regions. Furthermore, an integrative analysis of DHS regions using TF chromatin immunoprecipitation sequencing (ChIP-seq) from the ENCODE project confirmed our *in silico* results. Combining these results, the discovery of the introduction of divergent motifs across evolutionary time highlights the co-evolution between TF binding specificities and the functional effects of *cis*-regulatory variants on gene expression, and therefore on phenotypic evolution.

## Materials and Methods

### Motif Prevalence Index

The primary TF binding motifs of humans and 73 other metazoan species were obtained from the Cis-BP database (Weirauch et al., 2014). Given a motif *x*, *n* species *S_1...n_* possessing its corresponding TF families can be revealed based on the annotations in the Cis-BP database. We constructed a phylogenetic tree *T_s_* with time of divergence between the 74 metazoan species based on the TimeTree database (Hedges et al., 2006) with neighbor-joining method, using the APE package of R (Paradis et al., 2004). Next, we used the species that had motif *x*, according to the Cis-BP annotation, to obtain subtree *T_x_*. It should be noted that *B(T_s_)* was the total length of branches in *T_s_*, and *B(T_x_)* was the sum of the lengths of all the branches from their common ancestor node to *n* species that had motif *x*. The motif prevalence index (MPI), which we defined as the ratio *B(T_x_)/B(T_s_)* and is a score between 0 and 1, was then calculated (Supplementary Figure S1). To obtain a reliable TF set for the motif-scanning analysis, we selected 364 motifs that were well-curated TF models from the JASPAR 2018 database (Khan et al., 2017). We used Tomtom (Gupta et al., 2007) to group them into 93 clusters of nonredundant motifs with a threshold *p*-value of < 0.05, and retained the motifs possessing the highest MPI in each cluster were retained.

### Identification of TF Binding Sites for Each Motif in Open and Closed Chromatin Regions

The human genome sequence and gene annotations were obtained from Ensembl (GRCh37, release 75; Flicek et al., 2014). We identified the occurrences of TF binding sites in the promoter regions (-1 kb to +500 bp from the transcription start site) for each of the 93 nonredundant motifs by scanning TF sequence preference in position-weight-matrix (PWM) format, using Matrix-scan from the RSAT (Regulatory Sequence Analysis Tools) toolbox (Turatsinze et al., 2008). Of note, we applied the Matrix-scan with a threshold false discovery rate of < 10^-4^, which is a recommended stringent parameter for putative *cis*-regulatory elements detection (Turatsinze et al., 2008). DNase I hypersensitive-site (DHS) cluster data were downloaded from the UCSC genome browser (Karolchik et al., 2004) for 125 cell types identified by the ENCODE project (Thurman et al., 2012). DHS peaks were defined as open chromatin regions, and chromatin regions without overlapping DHS peaks were defined as closed chromatin regions.

### The Ages of Human Genes

The ages of human genes arising at different evolutionary times were identified by combining homolog clustering with phylogeny inference, as described in recent literature (Yin et al., 2016). Gene category 1 denoted Primates origin, i.e., the youngest genes; category 2 denoted Mammalia origin; category 3 denoted Vertebrata origin; category 4 denoted Metazoan origin; category 5 denoted Eukaryota origin; and category 6 denoted cellular-organism origin, i.e., the oldest genes.

### Identification of Enriched Functions Associated With DHS Regions

In order to investigate the functional annotation of the DHS regions with the many divergent motifs, we collected the longer DHS regions (300–400 bp in length) in the promoters of protein-coding genes before eukaryotic origin (categories 5 and 6) and computed their mean MPI scores in the DHS-edge regions. In assessing the proportions of divergent versus common motifs in the DHS regions, the 10th percentiles of the mean MPI scores for the DHS edges were considered divergent, while the 90th percentiles were considered common. The functional enrichment of the gene sets near the divergent or common DHS regions was performed using GREAT (Genomic Regions Enrichment of Annotations Tool; McLean et al., 2010), with the default parameters and all DHS regions of a similar length (300–400 bp) as the background. In particular, the GREAT web interface was used to automatically submit DHS regions and retrieve results for subsequent parsing.

### TF ChIP-Seq and Enhancer Datasets

The ChIP-Seq peaks of 243 TFs (Supplementary Table S1) in numerous cell lines were downloaded from the ENCODE Consortium (Roadmap Epigenomics Consortium et al., 2015) based on the genome hg19 assembly. For each TF, the tracks of the same cell lines were combined by retaining the overlapping base pairs with at least half of the tracks. Since the average length of the ChIP-seq peaks were longer (∼300 bp) than those of the TF binding motifs, we applied TF binding sites of 25 bp before and after the summits of the ChIP-seq peaks. Overlaps of genomic intervals with TF ChIP-seq peaks and human enhancer regions obtained from either FANTOM5 (Atlas of transcribed enhancers, Andersson et al., 2014) or VISTA Enhancer Browser (Visel et al., 2007) calculated using Bedtools.

### Expression Data for TFs

The expression profiles of the human TFs were collected from the Human Protein Atlas (HPA; Uhlén et al., 2015). Since the HPA divides all human-expressed genes into five categories, we here categorized the expression of TF genes in relatively general terms, as either ubiquitous expression or tissue-elevated expression. The categories ‘expressed in all tissues’ and “mixed” from the HPA were grouped as ubiquitous expression. The categories “tissue-enhanced,” “group-enriched,” and “tissue-enriched” from the HPA were grouped as tissue-elevated expression.

### Code Availability

The computer code that supports the findings of this study is available from Git-Hub, with the identifier doi: 10.5281/zenodo.1208608.

## Results and Discussion

### Motif Prevalence Index Estimates the Divergence Level of Motif Sequences

**FIGURE 1:**
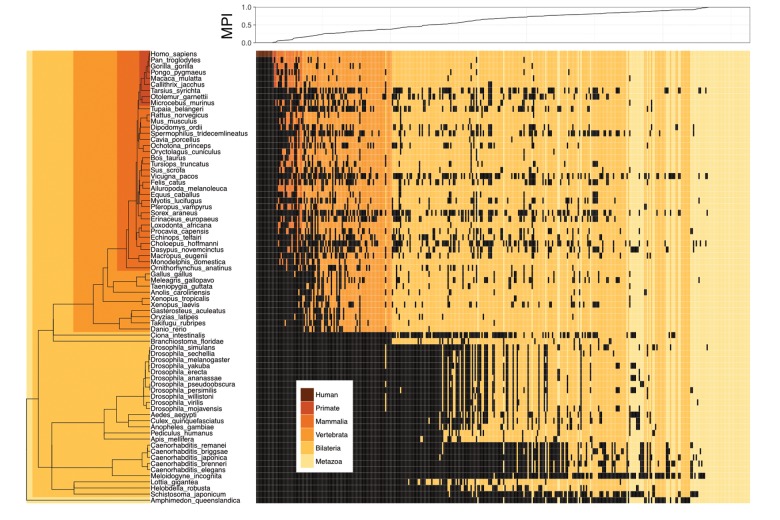
Motif prevalence index (MPI) of TF binding specificities in humans. Phylogenetic relationship between 364 human TF binding specificities (motifs) from the JASPAR database and their MPI scores (upper panel). Color codes denote the presence of motifs in various metazoan lineages. Black denotes the absence of motifs.

We proposed a new measure, the MPI, to estimate the evolutionary divergence level of TF DNA-binding preferences (motifs) in humans, based on the finding that the primary DNA-binding specificities of TFs with similar amino acid sequences in their DNA-binding domains (DBDs) are generally conserved between distantly related species (Jolma and Taipale, 2011; Weirauch et al., 2014; Nitta et al., 2015). Based on phylogenetic distance and the existence of a given motif (i.e., homologous TFs with conserved amino acid sequences in their DBDs, based on the *Cis-*BP database) across metazoan species, the MPI represented the evolutionary divergence level of human motifs, with a score from 0 (human-specific) to 1 (common in all 74 metazoan species used in this study). Next, we selected the human motifs for which there is experimental evidence in the JASPAR database (Khan et al., 2017). Most of the human motifs (72.8% of the 364 motifs shown in Supplementary Tables S1, S2) were common across the Metazoa and Bilateria taxa, but the divergent motifs (MPI < 0.1, 7.7%) in humans emerged approximately after the divergence of the Vertebrata lineage (Figure 1). The MPI was not biased by some intrinsic motif properties, such as motif length or information content (no significant correlation; Supplementary Figure S2), but the GC content was significantly lower in the more divergent motifs. Moreover, the finding that there was no significant correlation between the MPI and the gene ages of the corresponding TFs reflects the independence of their evolutionary history from the changes in their binding specificity of the TF repertoires.

### Edges of DHS Regions Prefer Divergent Motifs

**FIGURE 2:**
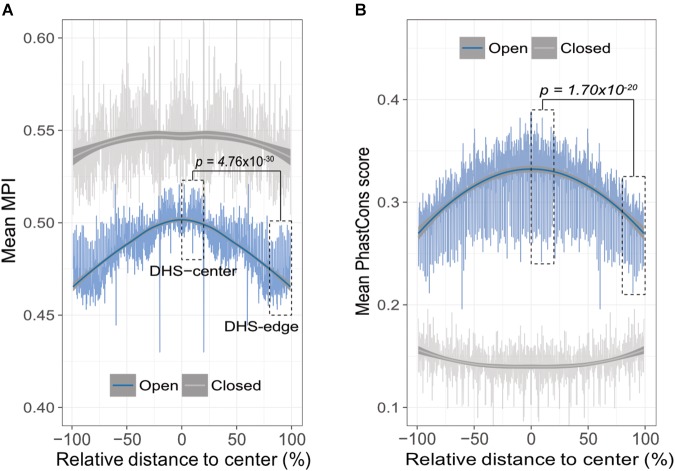
Edges of open-chromatin regions preferentially for the emergence of divergent TF binding motifs. **(A)** Distribution of mean MPIs at different relative positions within chromatin regions of 150–400 bp. Since DHS regions differ with respect to the lengths of their peaks, the mean MPI distribution for DHSs was calculated in 0.1% relative-distance sliding windows. The relative distance was defined as the normalized distance from the center of the fragments, ranging from 0% at the center to 100% at the edge of a given DHS peak. The mean MPI scores mirror each other around the center of the DHS regions. DHS-center denotes the quintile regions in the center of a DHS, and DHS-edge denotes the decile regions in both DHS borders. “Open” denotes the DHS regions and “closed” denotes the promoter regions without overlapping with DHSs. **(B)** Distribution of the mean PhastCons conservation scores at various relative positions within open- and closed-chromatin regions of 150–400 bp. *P*-values in **(A)** and **(B)** for comparisons between the DHS-center and -edge regions were obtained using one-sided Wilcoxon rank-sum tests.

A theoretical study has suggested that the emergence of newly evolved binding sites occurs preferentially in the DNA sequences bordering pre-existing TFBSs (Tuǧrul et al., 2015). Accordingly, we propose that the relatively common motifs are located around the centers of the open-chromatin regions, whereas relatively divergent motifs are located in the border regions. To test this hypothesis, we conducted an *in silico* motif scan from 1 kb upstream to 500 bp downstream of transcription start sites (TSS) in protein-coding genes, and further filtered the DNase I hypersensitivity-site (DHS) clusters in 125 cell types, that are highly corresponding to TFBSs (Thurman et al., 2012). We then investigated the open-chromatin regions, as defined by DHS peaks in the range 150–400 bp (i.e., one to two nucleosome-free regions), which theoretically contain several TFBSs, and then computed the mean MPI of the motifs that were identified. It is important to note that, to reduce the ambiguity of motif occurrences in similar motif patterns, we focused on 93 nonredundant JASPAR motifs that were clustered by Tomtom (Gupta et al., 2007), with a threshold *p*-value of < 0.05. The MPIs of these motifs remained evenly distributed (Supplementary Figure S3). As expected, the spatial distribution of the mean MPI scores decreased significantly from center to border within the DHS regions (Spearman’s correlation coefficient *rho* = -0.753, *p* < 2.2 × 10^-16^; Figure 2A). Specifically, the mean MPI scores in the DHS-edge zones (the decile regions of both DHS borders) were significantly lower than those in the DHS-center zones (the quintile regions of the center of the DHS; one-sided Wilcoxon rank-sum test, *p* = 4.76 × 10^-30^; Figure 2A). In contrast, the closed-chromatin regions in the promoters showed a negligible decline in their mean MPI scores (Spearman’s correlation coefficient *rho* = -0.01; Figure 2A), and these were similar to the mean values obtained by randomly selecting a subset of 93 nonredundant motifs 1000 times (Supplementary Figure S4). Additionally, we noted a significantly decreasing correlation between motif MPIs and the occurrence ratios of open-to-closed chromatin regions (Supplementary Figure S5). In other words, one likely explanation for the lower mean MPI scores in the open-chromatin regions is that divergent motifs arise preferentially in these regions. Since the divergent motifs with lower MPIs are the TFs that have evolved to recognize new DNA sequences across evolutionary time, the question immediately arose as to whether the DNA sequences in the DHS regions exhibit different conservation levels.

Thus, we sought to determine whether the decreasing trend in mean MPI as a function of position was systematically paralleled by changes of evolutionary conservation in open-chromatin regions. We used the PhastCons score (Siepel et al., 2005) to calculate the levels of evolutionary conservation of DNA sequences from alignments of 99 vertebrate genomes (Rosenbloom et al., 2015). As expected, the open-chromatin regions (DHSs) possessed higher conservation levels than the closed-chromatin ones, which have the highest background mutation rate (Prendergast et al., 2007; Figure 2B). In fact, the flattened distribution of the mean MPI scores of the closed-chromatin regions without evolutionary constraint could be the result of randomly introduced motifs across the regions. However, the PhastCons scores in the DHS-center zones of the open-chromatin regions were significantly higher than those in the DHS-edge zones (one-sided Wilcoxon rank-sum test, *p* = 1.70 × 10^-20^; Figure 2B). Of note, there was no correlation between MPI values of motifs and the mean PhastCons scores of their occurrences (Supplementary Figure S6), because the conservation in TF binding specificities and in the sequences of TFBSs were independently from each other. Therefore, a modest evolutionary constraint at the edges of the DHS regions is most likely to reflect the rapid TFBS turnover, which would readily allow the introduction of divergent motifs.

### DHS Regions With Many Divergent Motifs at the Edge Are Associated With Specific Functions

**FIGURE 3:**
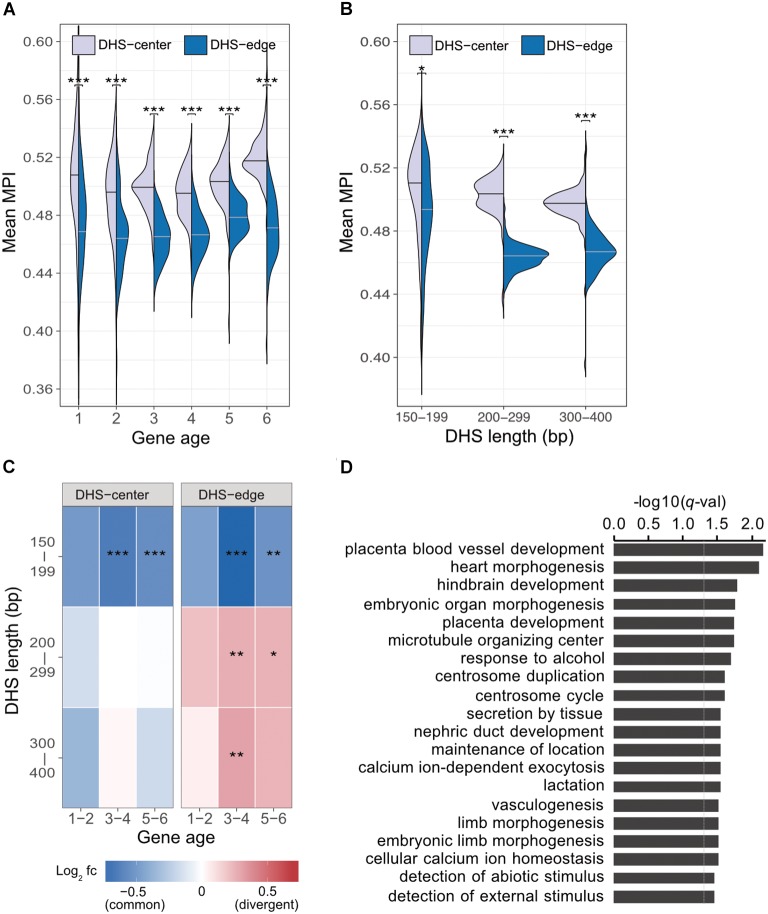
Motif enrichment in the promoter regions of protein-coding genes across different gene ages and DHS lengths. **(A)** Comparison of mean MPIs between DHS-center regions (purple, left violin plot) and DHS-edge regions (blue, right violin plot) in genes of six age categories. Age categories are as follows: (1) Primates origin (youngest genes), (2) Mammalia, (3) Vertebrata, (4) Metazoa, (5) Eukaryota; and (6) cellular organisms (oldest genes). **(B)** Differences in mean MPI between DHS-center and -edge regions, stratified according to DHS length. Significant values in **(A)** and **(B)** were obtained using the Wilcoxon rank-sum test after applying a Bonferroni correction for multiple tests. **(C)** Enrichment of motif occurrences. The color of the cells indicates the fold-changes (log_2_ *fc*) of occurrences of divergent motifs divided by common motifs. Divergent motifs were defined as those with MPI < 0.1, and common motifs as MPI ≥ 0.9. Fisher’s exact test was used to examine whether the proportion was significantly different (2 × 2 contingency table, in which rows correspond to occurrences inside/outside of a section of a DHS, and columns represent TF groups). Significant values were obtained after the Bonferroni correction for multiple tests had been applied. ^∗^*p* < 10^-2^; ^∗∗^ *p* < 10^-3^; ^∗∗∗^ *p* < 10^-4^. **(D)** Results of GREAT functional annotation of the longest DHS regions with many divergent motifs in their boundary regions. The -log_10_ of hyper FDR *q*-values is reported.

Previous studies indicate that regulatory complexity, such as the number of TFs regulating a gene, increases continuously over evolutionary time (Warnefors and Eyre-Walker, 2011; Berthelot et al., 2018). We thus examined whether the differences between the mean MPI scores for the DHS-center and -edge regions were constant across genes of different ages. We found that there was a consistent significant difference for the promoters of protein-coding genes of all ages (Figure 3A). Despite this, there were larger numbers of longer DHSs in the older genes (Supplementary Figure S7). We then performed a further analysis (Figure 3B) incorporating DHS length as a variable, and found that the differences between the DHS-center and -edge regions were greater for the longer DHSs (> 200 bp). Intrigued by these results, we compared the fold enrichment of the motif occurrences between divergent (MPI < 0.1) and common motifs (MPI ≥ 0.9) across gene ages and DHS lengths. The divergent motifs were not enriched in the short DHS (150–199 bp) regions, but were in the boundary regions of longer DHSs (Figure 3C). Similar robust results were found when applying different cut-offs for specific (MPI < 0.2) and common motifs (MPI ≥ 0.8) (Supplementary Figure S8). Therefore, one feasible interpretation of our observations is that the introduction of divergent motifs is likely to accompany the elongation of *cis*-regulatory DNA regions, particularly on the boundaries.

We next explored whether longer DHS regions with many divergent motifs in their edge regions were associated with genes for specific biological functions. We analyzed the longer DHSs (300–400 bp) in the promoters of older genes (groups 5 and 6) and found larger numbers of the DHSs displaying low mean MPI scores at their edge regions (Supplementary Figure S9). We used GREAT (McLean et al., 2010) to determine the associated functions of the gene sets found in the proximity of DHS regions with many divergent motifs (10th percentiles of the mean MPI scores at the edges) or many common motifs (90th percentiles). Unexpectedly, those neighboring DHS regions with many common motifs at the edges were not associated with any functions. However, those DHS regions with many divergent motifs at the edges were linked to genes showing significantly enriched functions in biological processes related to morphogenesis and development, such as heart morphogenesis (GO:0003007, *q*-val = 7.98 × 10^-3^) and placenta blood-vessel development (GO:0060674, *q*-val = 6.82 × 10^-3^; Figure 3D, and full results in Supplementary Table S3). With an increased number of longer DHSs in the promoters of older genes, therefore, such expansion of *cis*-regulatory regions via the introduction of divergent motifs could contribute to the regulatory complexity of genes related to tissue development across evolutionary time.

### TF ChIP-Seq Reveals Similar Distribution of MPI Scores Within DHS Regions

**FIGURE 4:**
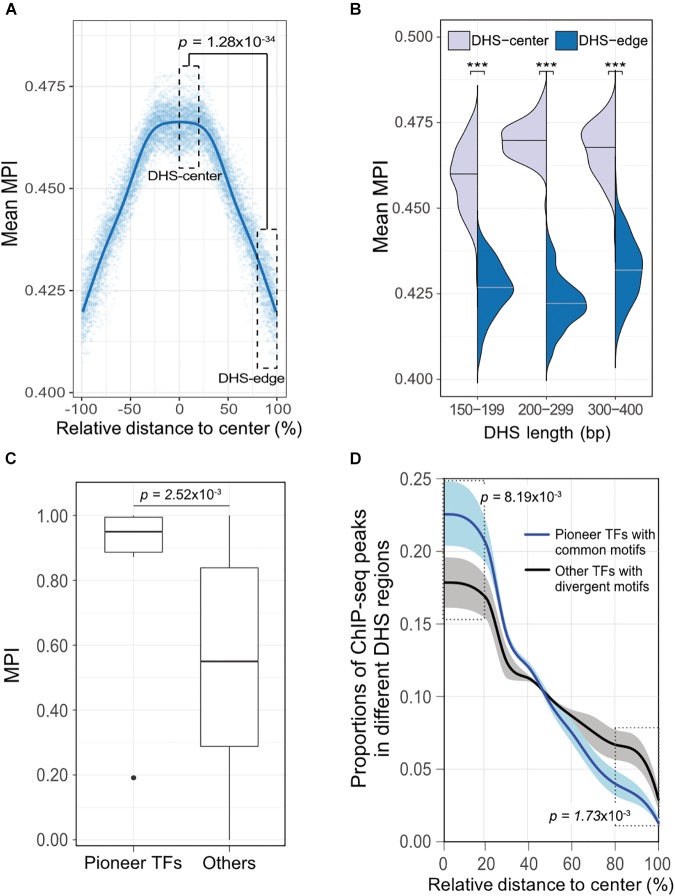
The differential preference of *cis*-regulatory regions harboring TF binding motifs in the human genome. **(A)** Distribution of mean MPIs for different relative positions within DHSs, based on the overlapped ChIP-seq peaks of 243 TFs with genome-wide DHS regions of 150–400 bp. The mean MPI scores mirror each other around the centers of the DHS regions. *P*-value for comparison between the DHS-center and -edge regions was obtained using a one-sided Wilcoxon rank-sum test. **(B)** Differences in mean MPI between DHS-center and -edge regions, stratified by DHS length. Significance of differences was assessed using one-sided Wilcoxon rank-sum tests followed by Bonferroni correction. ^∗^*p* < 10^-2^; ^∗∗^*p* < 10^-3^; ^∗∗∗^*p* < 10^-4^. **(C)** Differences in the MPIs of motifs corresponding to pioneer TFs and other motifs. *P*-value was obtained using a one-sided Wilcoxon rank-sum test. **(D)** The proportions of different TF–ChIP-seq signals in different DHS regions. Pioneer TFs with common motifs (MPI = 1) were FOXA1, FOXA2, RFX1, RFX3, and RFX5; other TFs with divergent motifs (MPI = 0) were NRF1, ZBED1, and ZBTB33. Data are the averaged proportions of ChIP-seq peak signals that overlapped with DHS regions for pioneer TFs and other TFs. Shaded areas show the standard deviation of the average across TFs. *P*-values were obtained using *t*-tests for the difference between pioneer TFs and other TFs in the DHS-center and -edge regions, respectively.

To validate our discovery of the motif distribution within the *cis*-regulatory DNA regions independently of the motif-scanning approach, we overlapped DHSs using *in vivo* chromatin immunoprecipitation followed by DNA sequencing (ChIP-seq) data. We used 243 TFs (Supplementary Table S1) downloaded from the ENCODE project (Wang et al., 2012), and recalculated the mean MPI scores using the corresponding MPIs of the TFs. Remarkably, the empirical TF–ChIP-seq results for within-DHS region means revealed significantly lower mean MPI scores for the borders than the central regions, on a genome-wide scale (Figure 4A, Spearman’s correlation coefficient *rho* = -0.940, *p* < 2.2 × 10^-16^). This result was highly consistent with the *in silico* motif-scanning results (Figure 2A). Additionally, the differences in mean MPI between DHS-center and -edge regions were significantly different among several *cis*-regulatory regions, such as gene promoters (protein-coding genes, non-coding genes, and pseudogenes) and enhancers, which were obtained from either FANTOM5 (Andersson et al., 2014) or VISTA (Visel et al., 2007) (Supplementary Table S4). The TF–ChIP-seq results also confirmed that the significant differences in the mean MPI scores between DHS-center and -edge regions were consistent for DHSs of different lengths (Figure 4B).

Besides, we noticed that the motifs corresponding to those pioneer TFs that were reported for chromatin-remodeling activity (Vernimmen and Bickmore, 2015) had significantly higher MPIs than others (Figure 4C, one-sided Wilcoxon rank-sum test, *p* = 2.52 × 10^-3^). Such high MPIs for most pioneer factors implies that their binding specificities are highly conserved throughout metazoan species. Pioneer TFs have been recognized to disrupt chromatin structure to create a nucleosome-free DNA region, and in turn, allow other TFs to access the nearby DNA regions (Zaret and Carroll, 2011; Müller and Tora, 2014). Accordingly, we next sought to examine a hypothetical scenario that pioneer TFs prefer to locate in the middle of open-chromatin regions, using a direct assessment of their TF–ChIP-seq data. We also did a genome-wide comparison of the distribution of ChIP-seq signals in the DHS regions for pioneer TFs with common motifs (MPI = 1) or the other TFs with most divergent motifs (MPI = 0). We found that pioneer TFs were located mostly in the centers rather than in the edges of DHS regions (Figure 4D). In contrast, the TFs with divergent motifs showed a distinct distribution pattern, with more occurrences in the DHS-edge regions (Figure 4D). Hence, the binding preferences of pioneer TFs provide a feasible rationale to explain the higher mean MPI scores for the DHS-center regions.

In summary, our results for both the *in silico* motif scan and the experimentally derived TF–ChIP-seq analysis unveil a differential preference of TFBSs within *cis*-regulatory DNA regions, whereby the border regions tend to harbor motifs that are bound by TFs with divergent DNA-binding specificities.

### TFs With Divergent Motifs Tend to Be Ubiquitously Expressed in Human Tissues

**FIGURE 5:**
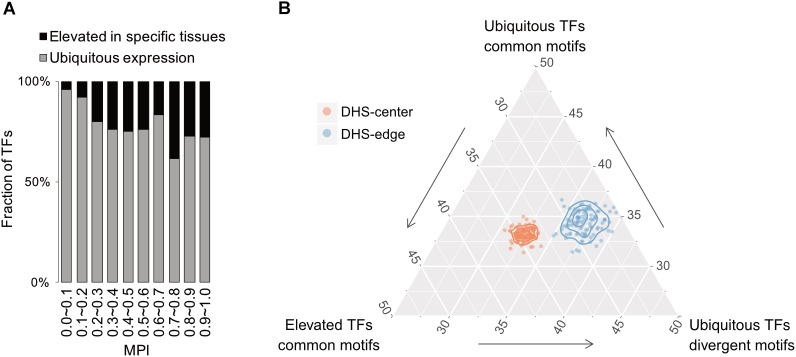
Relationship between motif divergence and tissue specificity of TFs. **(A)** The fraction of TFs with ChIP-seq data, classified according to their tissue expression pattern (Uhleìn et al., 2015) for each of the corresponding MPI ranges. Gray denotes a group of ubiquitous expression in human tissues and black denotes another group with elevated expression in specific tissues. **(B)** Ternary proportion distributions for the TF-ChIP occurrences in different DHS regions. The proportions were calculated as the fraction of each group of TF-ChIP-seq peaks in the given DHS regions. The ubiquitous TFs with divergent motifs (MPI < 0.1, ubiquitous expression) and the TFs with common motifs (MPI ≥ 0.9, ubiquitous expression or tissue-specific expression) were grouped as in (A). DHS-center denotes the quintile regions of the DHS, and DHS-edge denotes the decile regions in both DHS borders.

Based on expression profiles for 32 human tissues obtained from the Human Protein Atlas (HPA; Uhlén et al., 2015), we divided TFs into one group showing ubiquitous expression (that are expressed in most tissues) and another showing significantly elevated expression in at least one human tissue. Remarkably, the majority of TFs possessing divergent motifs are ubiquitously expressed in human tissues, whereas the larger numbers of TFs possessing common motifs, i.e., those with higher MPIs, are more strongly expressed in specific human tissues (Figure 5A for the TFs with ChIP-seq data, Supplementary Figure S10 for all other TFs from the HPA). Notably, a recent study has reported that duplicate genes tend to diverge in their expression profiles in different tissues during the course of evolution (Kryuchkova-Mostacci and Robinson-Rechavi, 2016). According to our observations, a common motif is usually shared by a couple of members of TF paralogs (Supplementary Table S1). The higher fraction of TFs showing tissue-specific expression most likely accounted for the larger number of gene paralogs. Thereafter, we computed the fold enrichment of the TF–ChIP-seq signals within the DHS regions by comparing the ubiquitously expressed TFs with divergent motifs (MPI < 0.1) with all TFs with common motifs (MPI ≥ 0.9). We found that the former were significantly enriched in the DHS-edge regions and represented a higher proportion of the total than the latter (Figure 5B and Supplementary Figure S11 for the enrichment analyses). In contrast, the tissue-specific TFs with common motifs represented the highest proportion of the total and were significantly enriched in the DHS-center regions (Figure 5B and Supplementary Figure S11). Taken together, these results provide the insight that DHS-center regions are bound by tissue-specific TF paralogs, which share similar motifs, while the DHS-edge regions are enriched in ubiquitously expressed TFs with divergent motifs. These results therefore imply that there is another level of transcriptional regulation dynamics affecting the interplay of DNA motifs and the distinct expression patterns of TFs.

Extensive studies indicate that the alternations of genomic sequences in TFBSs are widespread in metazoan species, even in closely related species (Villar et al., 2014). The patterns in our mean MPI scores, which correspond to different levels of divergence in TF binding specificity, indicate that the introduction of divergent motifs occurs preferentially in the borders of *cis*-regulatory regions (as opposed to their centers; Figures 2A, 4A). Our results are in line with theoretical studies, which show that sequences adjacent to ancestral TFBSs readily evolve, facilitating the emergence of new TFBSs (Payne and Wagner, 2014; Tuǧrul et al., 2015). Since common motifs (high MPIs) are prevalent among metazoan species, the central *cis*-regulatory regions are most likely to contain ancestral binding sites and to be constrained over evolutionary time, as indicated by their higher PhastCons scores (Figure 2B). Moreover, TFBS clustering in the genomic regions with the cooperative interactions of multiple regulators can be a consequence of fast turnover of genetic sequences for TF binding evolution (Tuǧrul et al., 2015; Khoueiry et al., 2017).

**FIGURE 6:**
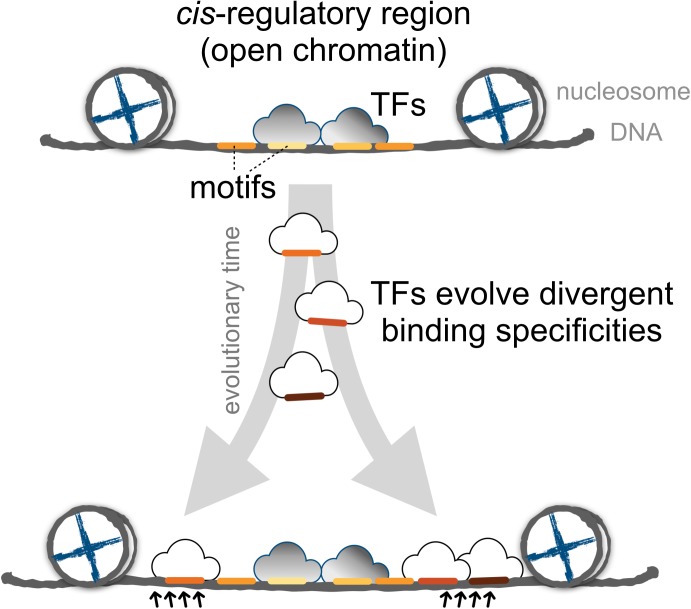
The proposed model for the dynamics of TF binding motifs in *cis*-regulatory regions. Divergent motifs occur preferentially in the borders of *cis-*regulatory regions across evolutionary time.

Finally, we proposed a model for the expansion of TFBSs with conserved motifs via the introduction of divergent motifs to adjacent sites in the *cis*-regulatory regions (Figure 6). *Cis-*regulatory evolution, such as changes in TFBSs over the evolutionary time scale, is an important source of diversity in the development of morphological traits via the gradual modification of transcription circuits (Levine and Tjian, 2003; Lynch and Wagner, 2008; Nocedal and Johnson, 2015). Studies on the effect of genetic variation on TF binding from ChIP-seq experiments provided direct evidence that the TF binding divergence is often a result of sequence changes in the bound genetic sequences (Schmidt et al., 2010; Reddy et al., 2012; Stefflova et al., 2013). Furthermore, TFs often bind cooperatively to sites adjacent to regulatory regions (Wray et al., 2003; Stefflova et al., 2013), the regulatory circuits, by coordinating alternative TFs, could diversify as the motifs in the TFBS-enriched border regions are replaced, allowing the expansion of new motifs. Since the rewiring of regulatory networks is crucial for the evolution of divergent expression patterns (Baker et al., 2012; Jarvela and Hinman, 2015), we suspect that an expansion mechanism that incorporates more divergent motifs in the boundaries of *cis*-regulatory regions serves as a common evolutionary intermediate in the rewiring process.

## Author Contributions

J-HH and H-KT conceived the idea, designed the study, and wrote the manuscript. J-HH, RK, T-CL, and ZT developed the computational algorithms and performed the bioinformatics analysis. ZT provided guidance in data analysis and interpretation of the results. All authors contributed to amending the manuscript and have read the submitted version.

## Conflict of Interest Statement

The authors declare that the research was conducted in the absence of any commercial or financial relationships that could be construed as a potential conflict of interest.
