# Supplementary material for: Borders of Cis-Regulatory DNA Sequences Preferentially Harbor the Divergent Transcription Factor Binding Motifs in the Human Genome
Source: Front Genet. 2018 Nov 22;9:571. doi: 10.3389/fgene.2018.00571 (PMC6261980; doi:10.3389/fgene.2018.00571)
Supplement: Supplementary file 1 [file Data_Sheet_1.PDF]

Borders of *cis*-regulatory DNA sequences preferentially harbor the divergent transcription factor binding motifs in the human genome

Jia-Hsin Huang[1,†], Ryan Shun-Yuen Kwan[1,†], and Zing Tsung-Yeh Tsai[2], Tzu-Chieh Lin[1], Huai-Kuang Tsai[1,*]

[1]Institute of Information Science, Academia Sinica, Nankang, Taipei, 115, Taiwan

[2]Department of Computational Medicine and Bioinformatics, University of Michigan, Ann Arbor, 48109, MI, USA

[†] The authors wish it to be known that, in their opinion, the first two authors should be regarded as joint First Author

*** Correspondence:**
Huai-Kuang Tsai
hktsai@iis.sinica.edu.tw

Supplementary Figures

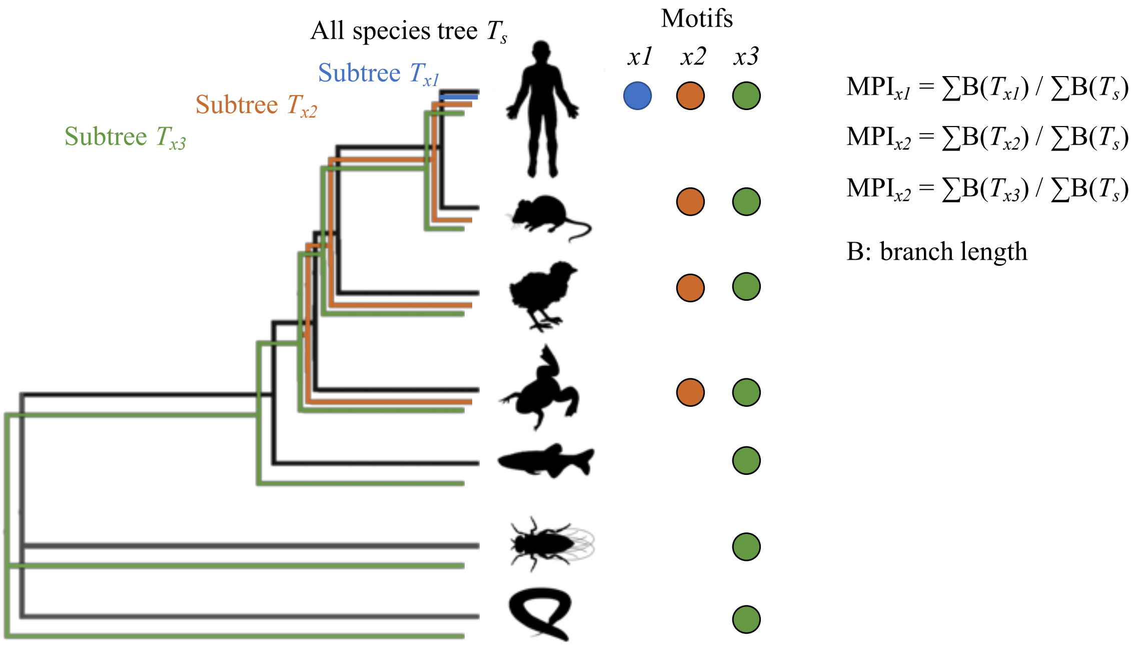

Supplementary Figure S1. A schematic of calculating motif prevalence index (MPI). The MPI for each motif x is the ratio $B(T_x)/B(T_s)$, *i.e.* the sum of branch lengths of a subtree divided by the total length of branches in the all species phylogenetic tree.

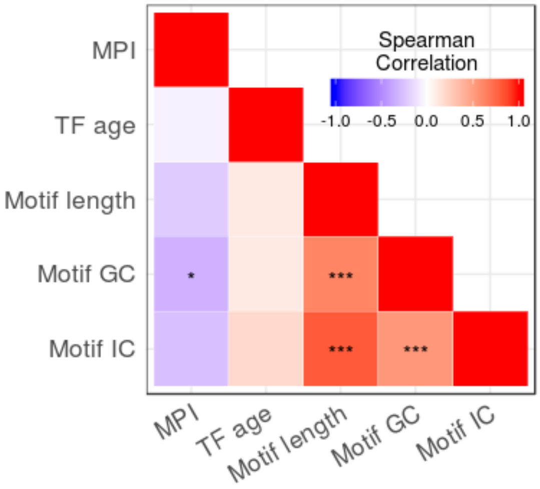

Supplementary Figure S2. A heatmap of the Spearman's correlation coefficients between pairs of features. * denotes the significant $p < 0.05$ and *** denotes the significant $p < 10^{-4}$.

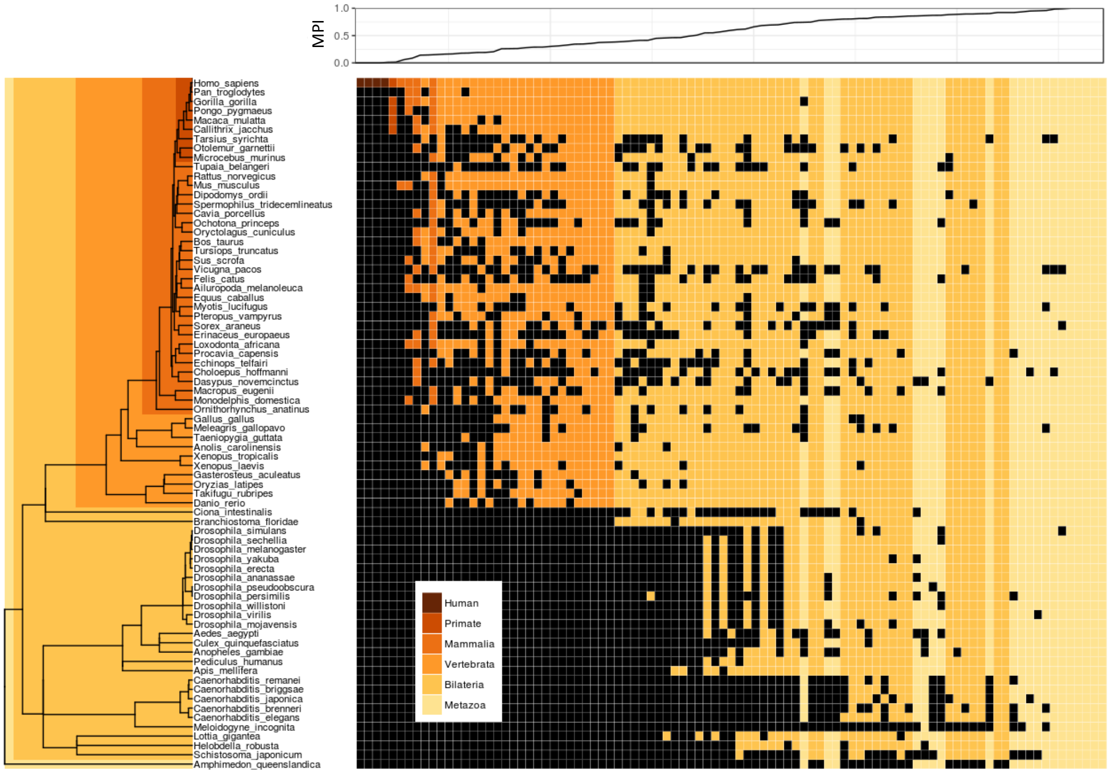

Supplementary Figure S3. Motif prevalence index (MPI) of non-redundant TF binding specificities in humans. Phylogenetic relationship of 93 clusters of non-redundant human TF binding specificities (motifs) clustered by Tomtom and their MPI scores (upper panel). Color codes denote the presence of motifs in various metazoan lineages. Black denotes the absence of motifs.

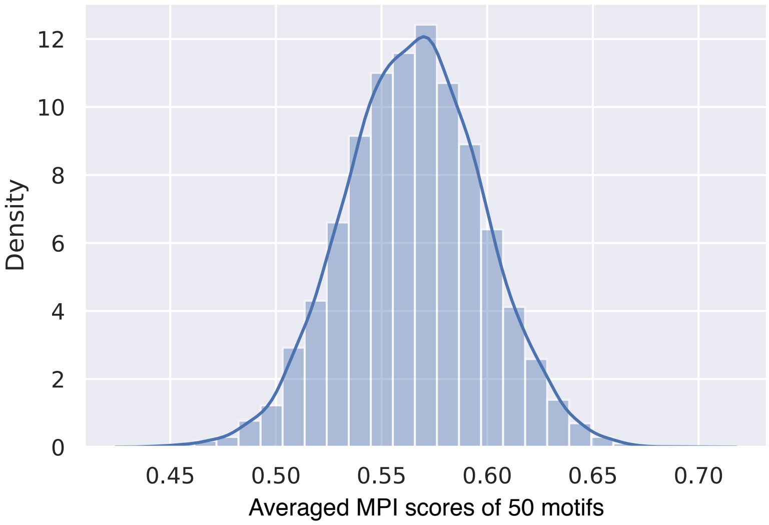

Supplementary Figure S4. Distribution of averaged MPI scores by randomly selected 50 motifs for 1,000 times. The mean value of the averaged MPI scores is 0.5653.

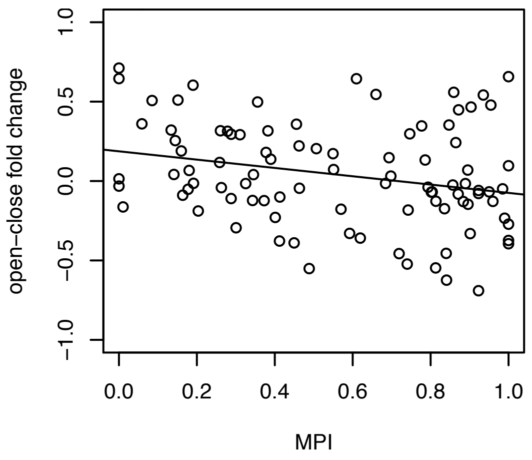

Supplementary Figure S5. Correlation between motif MPIs and the enrichment of motif occurrences in the open chromatin regions. The fold changes were the motif occurrences in the open chromatins divided by those in the closed chromatins in the promoter regions of protein-coding genes. Spearman correlation coefficient is -0.262 and *p*-value is 0.011.

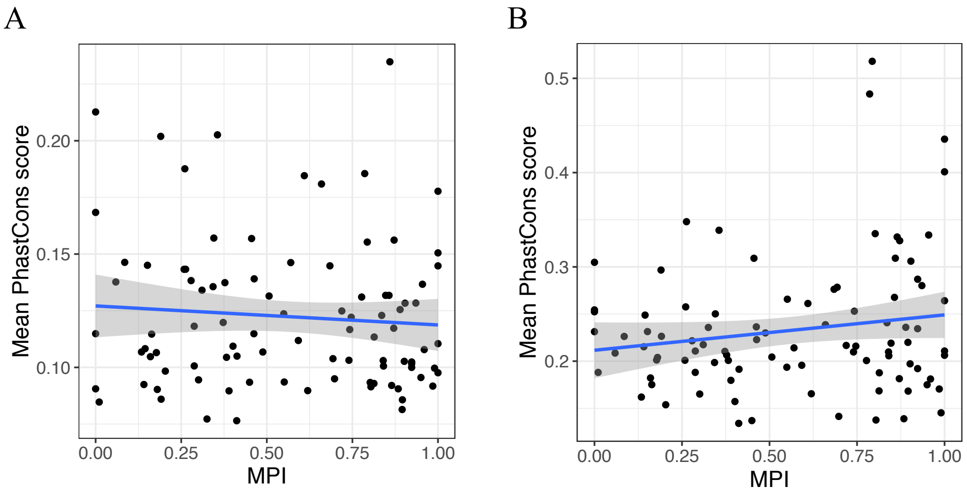

Supplementary Figure S6. Relationship between motif MPIs and the mean PhastCons scores of the motif occurrences in the promoter regions. There were no significant correlations in (A) open chromatin regions (Spearman correlation, $r = -0.08$, $p = 0.447$) and (B) closed chromatin regions (Spearman correlation, $r = 0.081$, $p = 0.439$) respectively.

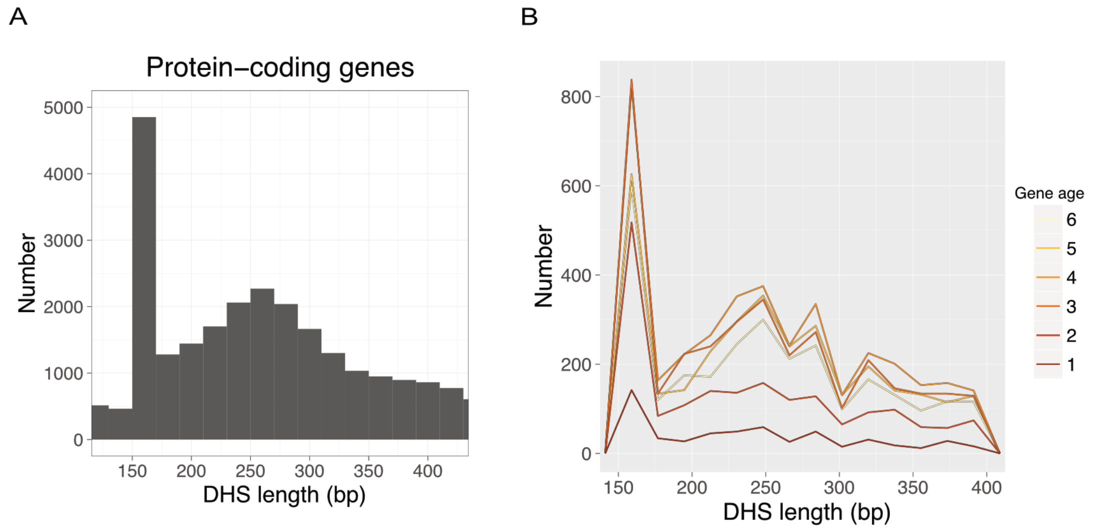

Supplementary Figure S7. Distribution of DHS lengths in the promoter of genes. (A) The number of DHS peaks across lengths in the promoter regions of the protein-coding genes. (B) The number of DHS lengths in the promoter regions of different ages of genes.

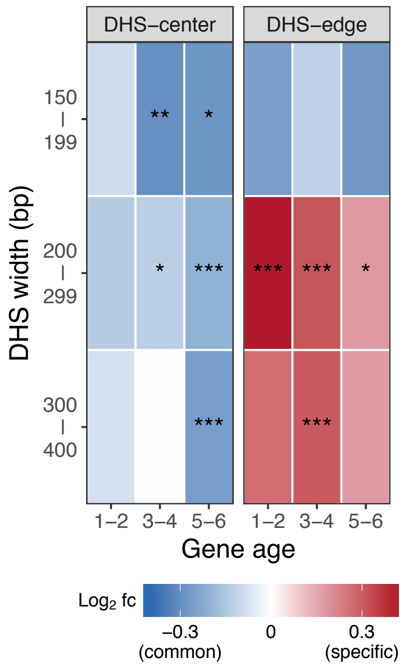

Supplementary Figure S8. Enrichment for motif occurrences. Color code in the cells indicates the fold-changes (log2) of occurrences for divergent motifs divided by common motifs. Specific motifs were MPI < 0.2; common motifs were MPI ≥ 0.8. Fisher's exact test was applied to examine whether the proportion was significantly different. Significant values were obtained after Bonferroni correction for multiple tests. *: $p < 10^{-2}$, **: $p < 10^{-3}$, ***: $p < 10^{-4}$.

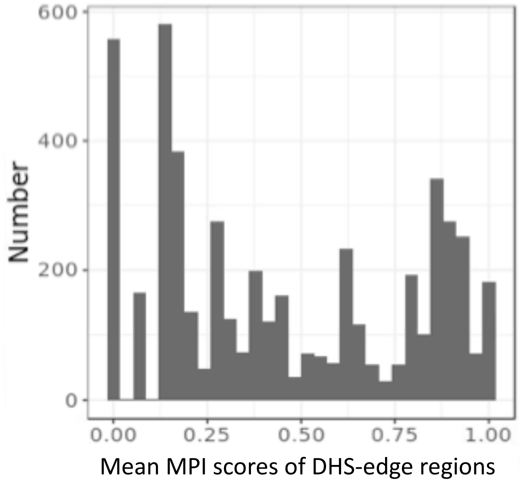

Supplementary Figure S9. Distribution of the mean MPI scores for the edge regions of long DHS peaks. The length of DHS peaks were 300–400 bp and presented in the promoters of older genes (categories 5 and 6 as shown in the Figure 3A).

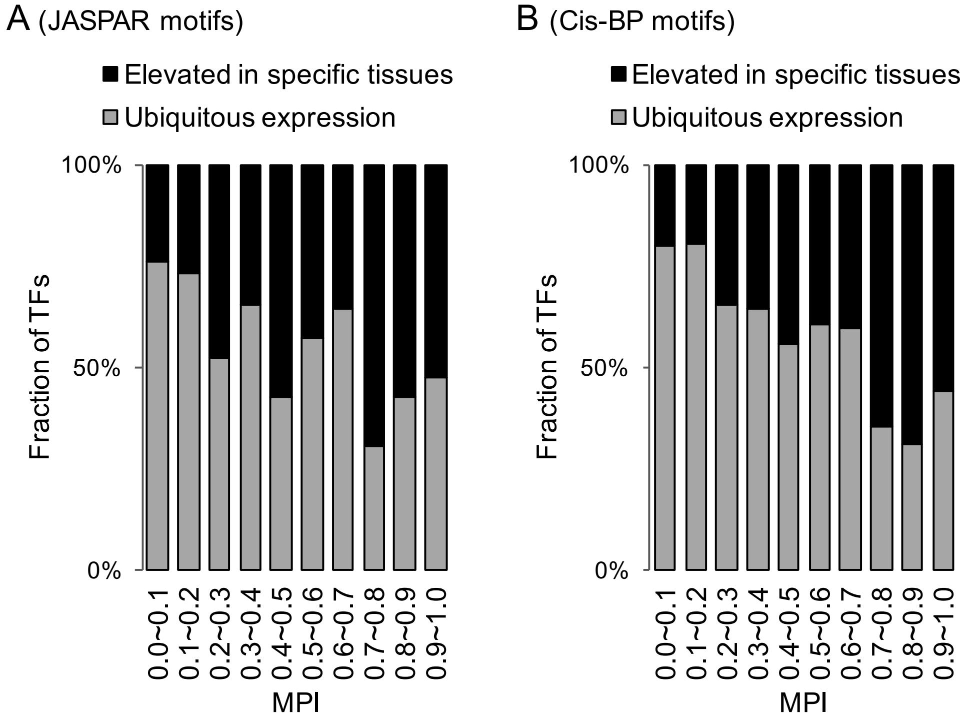

Supplementary Figure S10. The fraction of the TFs classified according to the tissue expression pattern (Uhlén et al., 2015) for each of corresponding MPI ranges. (A) The motifs of TFs are obtained from the JASPAR database (Khan et al. 2017). (B) The motifs of TFs are obtained from Cis-BP database (Weirauch et al. 2014). Grey denotes the TFs with ubiquitous expression in most human tissues and black denotes the TFs with an elevated expression specifically in at least one human tissue.

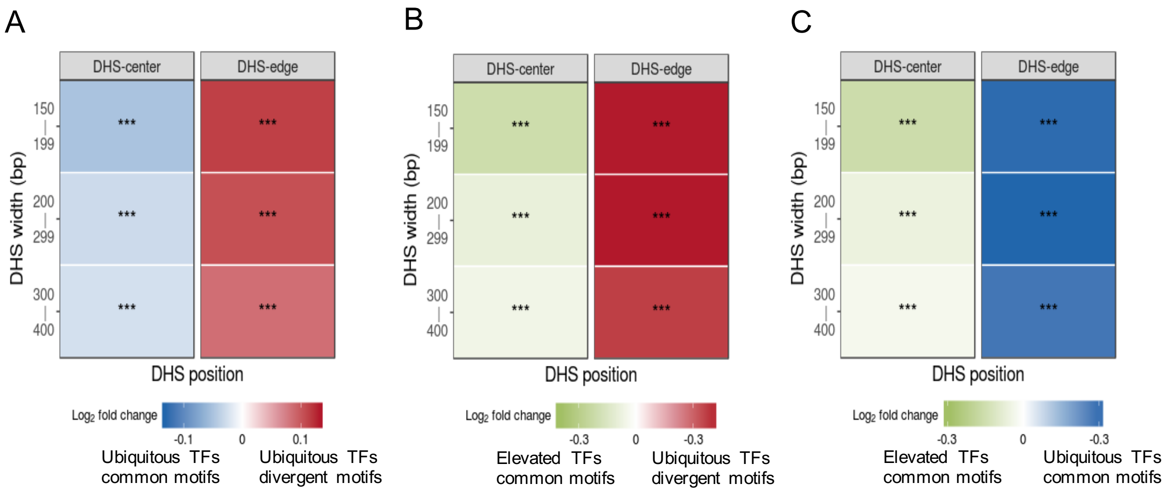

Supplementary Figure S11. Pair-wise enrichment tests for TF-ChIP occurrences overlapped with DHS regions comparing different group of TFs. The ubiquitous TFs with divergent motifs (MPI < 0.1, ubiquitous expression) and the TFs with common motifs (MPI ≥ 0.9, ubiquitous expression or tissue-elevated expression) were grouped as in Fig. 3C. Color codes in the cells (A, B, C) indicate the fold-changes (log2) of TF-ChIP occurrences between two groups. Fisher's exact test was applied to examine whether the proportion was significantly different. Significant values were obtained after Bonferroni correction for multiple test. ***: $p < 10^{-4}$.
