# Supplementary material for: Borders of Cis-Regulatory DNA Sequences Preferentially Harbor the Divergent Transcription Factor Binding Motifs in the Human Genome
Source: Front Genet. 2018 Nov 22;9:571. doi: 10.3389/fgene.2018.00571 (PMC6261980; doi:10.3389/fgene.2018.00571)
Supplement: Supplementary file 3 [file Data_Sheet_2.PDF]

Supplementary Table S2. The information of 364 human motifs from JASPAR database.

No.	TF	MPI	DBD family	Non redundant	Logo
1	HESX1	0	Homeobox	0	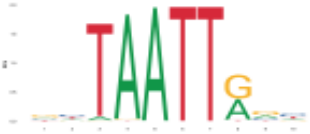
2	IRF7	0	IRF	0	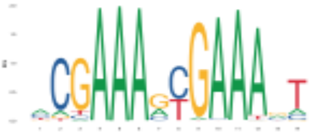
3	IRF8	0	IRF	1	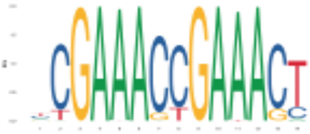
4	IRF9	0	IRF	0	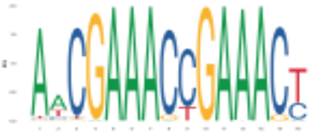
5	TP63	0	P53	0	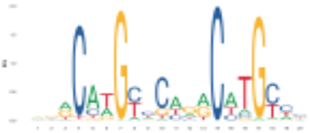
6	NFKB1	0	RHD	0	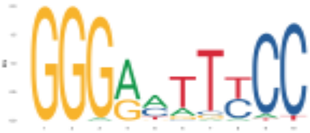
7	RUNX1	0	Runt	0	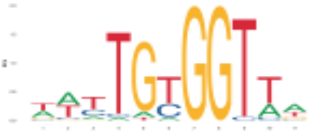
8	TEAD1	0	TEA	0	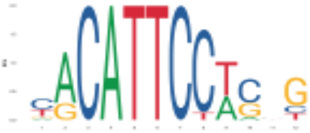
9	NFYB	0	UNKNOWN	0	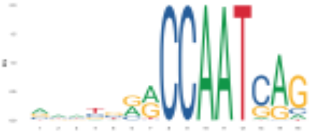
10	NRF1	0	UNKNOWN	1	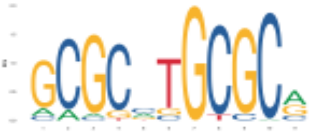

No.	TF	MPI	DBD family	Non redundant	Logo
11	ZBED1	0	UNKNOWN	1	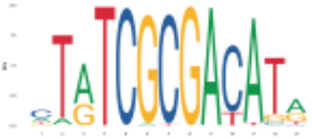
12	ZBTB33	0	zf−C2H2	1	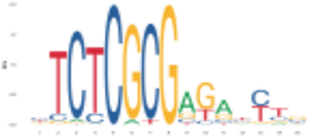
13	SRY	0.00099	HMG_box	0	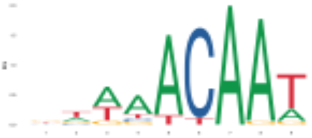
14	RHOXF1	0.0105	Homeobox	1	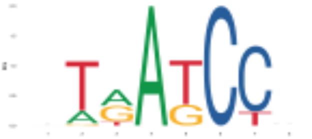
15	DUX4	0.01915	Homeobox	0	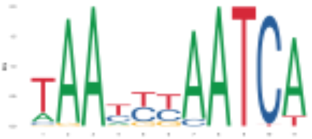
16	TFCP2	0.02054	CP2	0	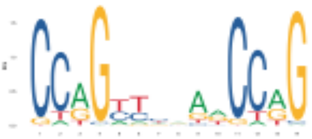
17	VENTX	0.05073	Homeobox	0	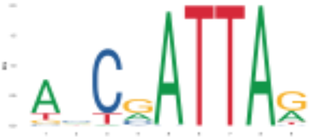
18	INSM1	0.05894	zf−C2H2	1	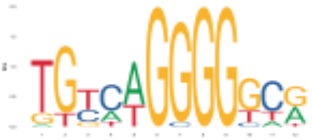
19	FOXP3	0.06456	Fork_head	0	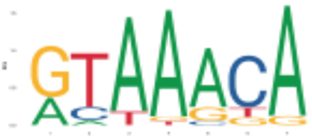
20	IRF2	0.06728	IRF	0	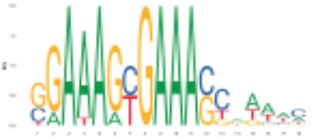

No.	TF	MPI	DBD family	Non redundant	Logo
21	IRF1	0.06852	IRF	0	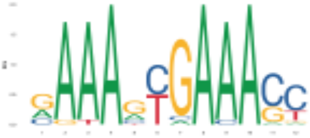
22	ATF7	0.07181	bZIP_1	0	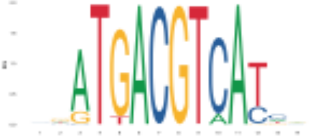
23	CENPB	0.07456	CENP−B_N	0	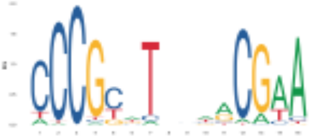
24	ZNF354C	0.0751	zf−C2H2	0	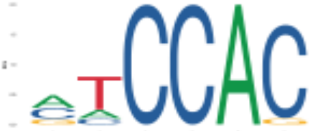
25	MZF1	0.0777	zf−C2H2	0	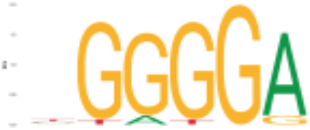
26	DUXA	0.07981	Homeobox	0	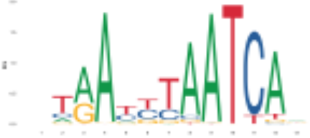
27	THAP1	0.08498	THAP	1	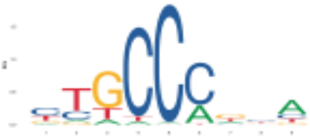
28	NOTO	0.08578	Homeobox	0	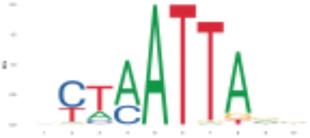
29	TEAD4	0.1289	TEA	0	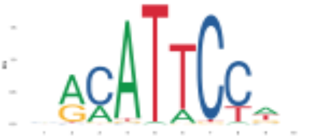
30	EBF1	0.13397	HLH	1	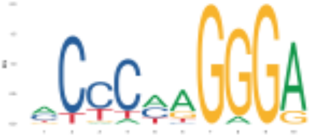

No.	TF	MPI	DBD family	Non redundant	Logo
31	E2F6	0.13809	E2F_TDP	0	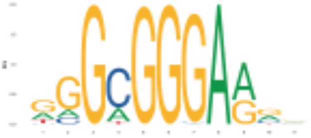
32	PROX1	0.14103	Prox1	1	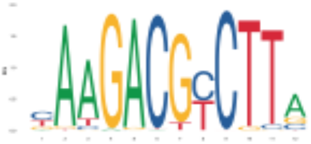
33	ZNF263	0.14457	zf−C2H2	1	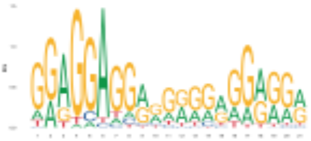
34	PAX4	0.14892	Homeobox,PAX	0	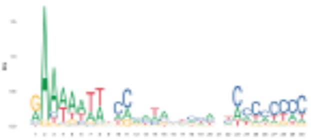
35	HINFP	0.15145	zf−C2H2	1	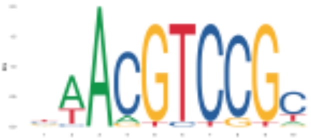
36	RREB1	0.16	zf−C2H2	1	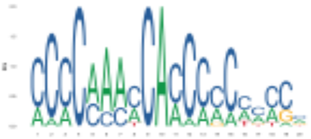
37	BCL6B	0.16368	zf−C2H2	1	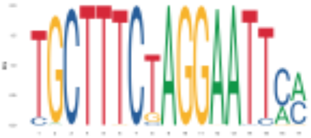
38	MGA	0.16621	HLH,T−box	0	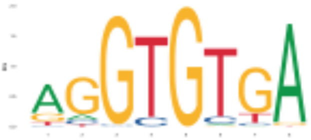
39	TEAD3	0.17745	TEA	1	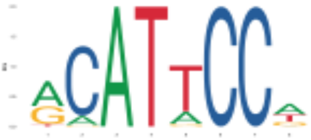
40	HIC2	0.18015	zf−C2H2	1	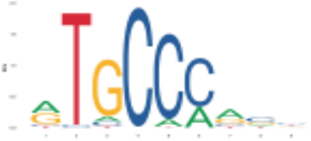

No.	TF	MPI	DBD family	Non redundant	Logo
41	ZBTB7B	0.18771	zf−C2H2	0	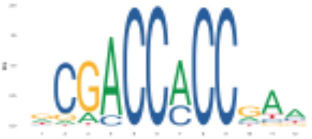
42	CTCF	0.19015	zf−C2H2	1	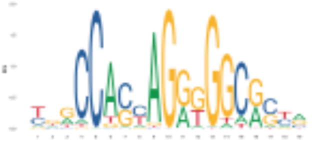
43	TP53	0.19135	P53	1	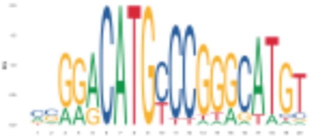
44	HMBOX1	0.20354	Homeobox	1	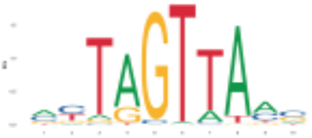
45	MYBL2	0.20611	Myb_DNA−binding	0	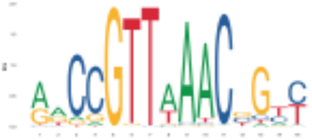
46	ESX1	0.2098	Homeobox	0	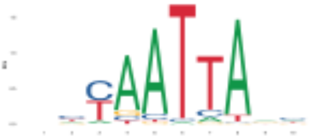
47	POU5F1B	0.22338	Homeobox,Pou	0	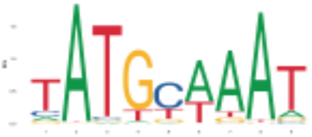
48	GMEB2	0.22809	SAND	0	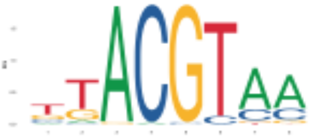
49	MIXL1	0.22849	Homeobox	0	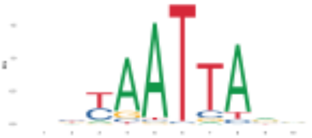
50	HES7	0.24249	HLH	0	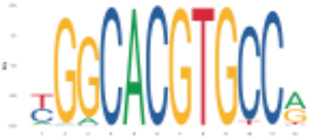

No.	TF	MPI	DBD family	Non redundant	Logo
51	E2F8	0.25074	E2F_TDP	0	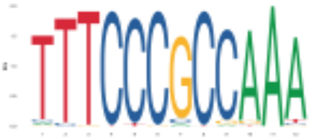
52	BATF3	0.25722	bZIP_1	0	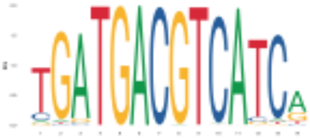
53	MYBL1	0.25842	Myb_DNA−binding	1	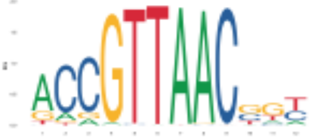
54	MLXIPL	0.25998	HLH	0	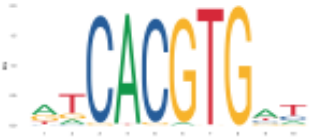
55	REST	0.26055	zf−C2H2	1	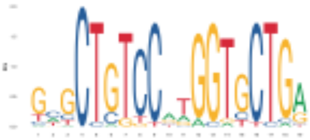
56	FIGLA	0.26167	HLH	0	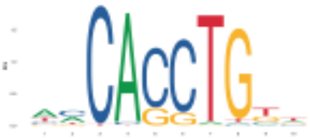
57	HES5	0.26192	HLH	0	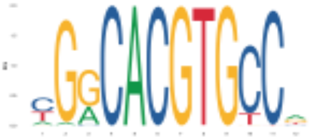
58	E2F7	0.26275	E2F_TDP	0	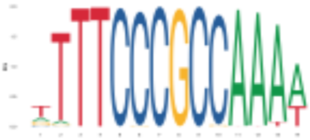
59	ZEB1	0.26284	zf−C2H2,Homeobox	1	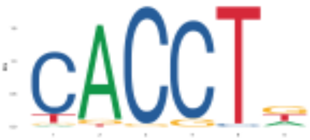
60	GLIS2	0.27231	zf−C2H2	0	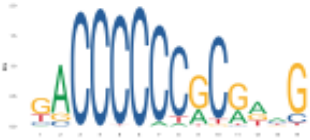

No.	TF	MPI	DBD family	Non redundant	Logo
61	E2F2	0.27431	E2F_TDP	0	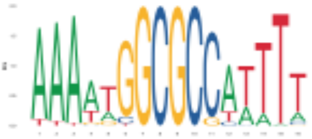
62	GLIS1	0.27447	zf−C2H2	0	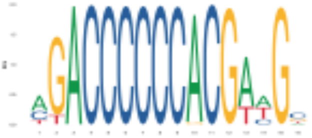
63	E2F1	0.27705	E2F_TDP	0	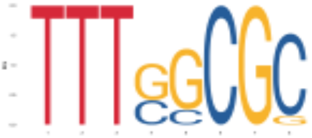
64	MTF1	0.27884	zf−C2H2	1	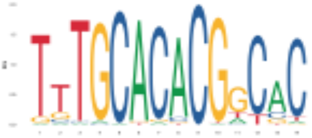
65	GRHL1	0.28793	CP2	1	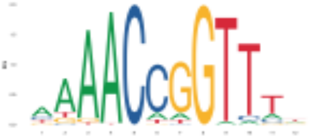
66	ZNF740	0.28807	zf−C2H2	1	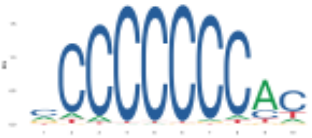
67	ZNF410	0.30064	zf−C2H2	1	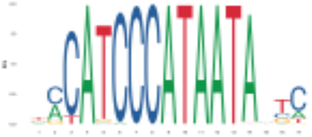
68	SOX4	0.30422	HMG_box	0	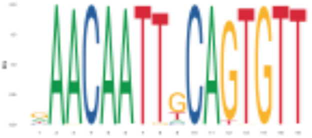
69	ZBTB7A	0.30498	zf−C2H2	0	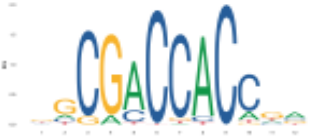
70	CUX2	0.30642	CUT,Homeobox	0	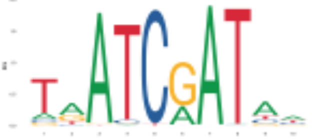

No.	TF	MPI	DBD family	Non redundant	Logo
71	ZBTB7C	0.31076	zf−C2H2	1	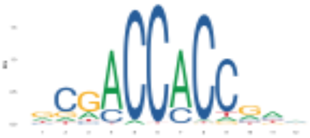
72	CUX1	0.31132	CUT,Homeobox	0	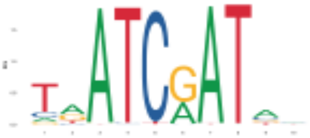
73	VAX1	0.31908	Homeobox	0	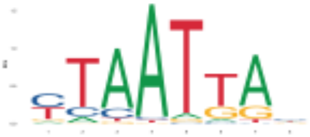
74	E2F3	0.32529	E2F_TDP	1	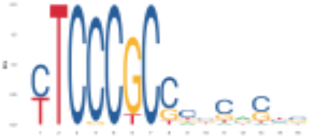
75	HSF4	0.32568	HSF_DNA−bind	0	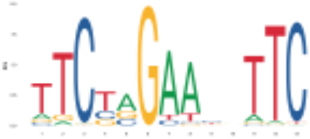
76	STAT1	0.32582	STAT_bind	0	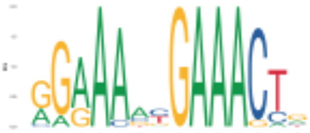
77	MEIS3	0.33177	Homeobox	0	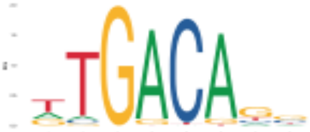
78	ATF4	0.3344	bZIP_1	0	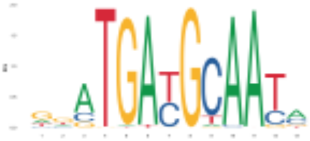
79	BHLHE40	0.33693	HLH	0	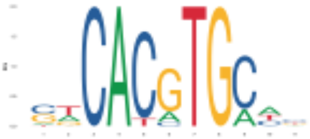
80	BHLHE41	0.33693	HLH	0	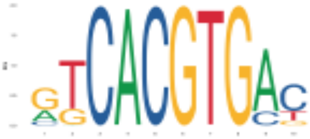

No.	TF	MPI	DBD family	Non redundant	Logo
81	HSF1	0.33851	HSF_DNA−bind	0	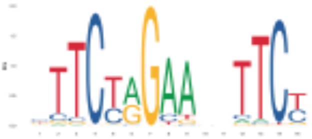
82	NFKB2	0.34133	RHD	0	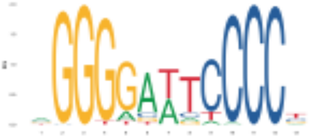
83	HSF2	0.34295	HSF_DNA−bind	1	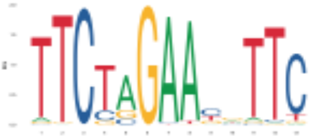
84	STAT3	0.34526	STAT_bind	1	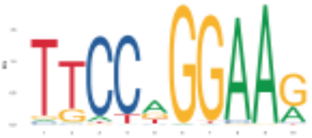
85	TP73	0.34677	P53	0	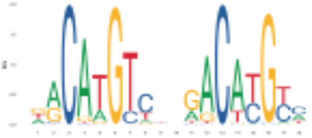
86	HOXC12	0.3489	Homeobox	0	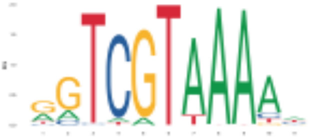
87	REL	0.35186	RHD	0	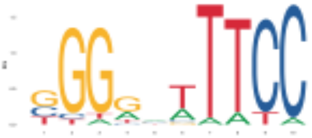
88	HOXD12	0.35446	Homeobox	0	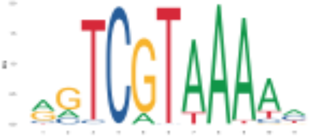
89	SPIB	0.35584	Ets	1	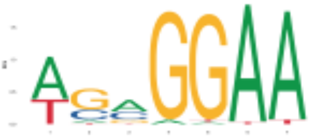
90	SPIC	0.36256	Ets	0	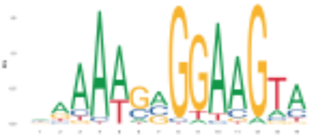

No.	TF	MPI	DBD family	Non redundant	Logo
91	LHX6	0.37209	Homeobox	0	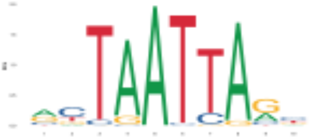
92	NFATC2	0.3726	RHD	0	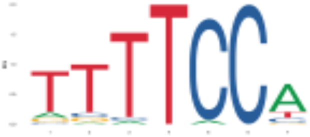
93	NFATC3	0.3726	RHD	1	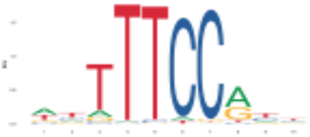
94	HOXA13	0.37309	Homeobox	0	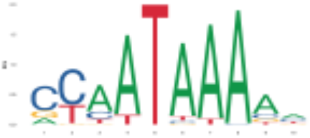
95	HOXD13	0.37309	Homeobox	0	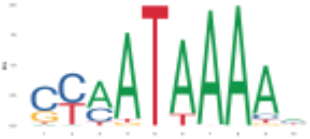
96	POU6F2	0.37322	Homeobox,Pou	0	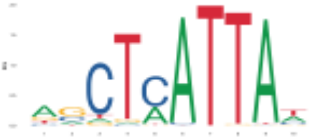
97	NFE2	0.37765	bZIP_1	0	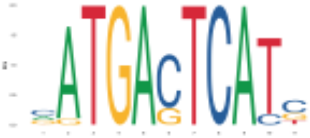
98	AR	0.37765	zf−C4	1	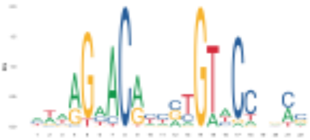
99	NR3C1	0.37765	zf−C4	0	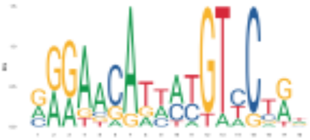
100	NR3C2	0.37765	zf−C4	0	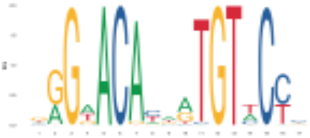

No.	TF	MPI	DBD family	Non redundant	Logo
101	PLAG1	0.38235	zf−C2H2	1	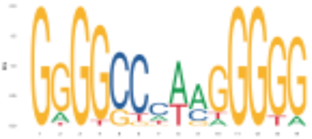
102	SOX10	0.3897	HMG_box	1	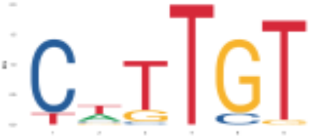
103	SOX9	0.3946	HMG_box	0	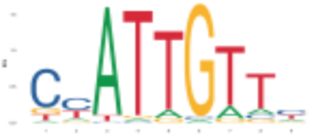
104	FOXH1	0.40121	Fork_head	1	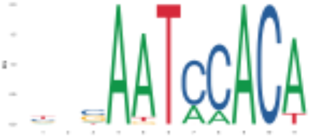
105	VAX2	0.40735	Homeobox	0	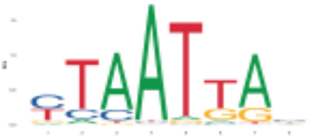
106	DMRT3	0.41206	DM	1	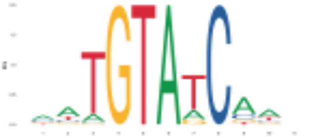
107	PRDM1	0.41295	zf−C2H2	1	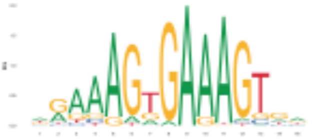
108	CEBPD	0.42614	bZIP_1	0	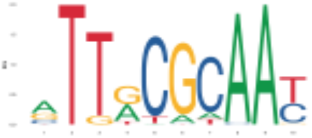
109	TBX21	0.43014	T−box	0	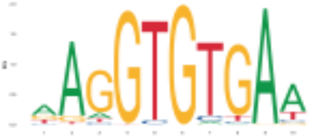
110	POU1F1	0.43483	Homeobox,Pou	0	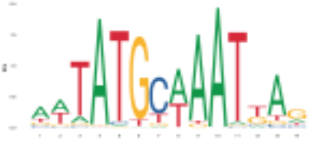

No.	TF	MPI	DBD family	Non redundant	Logo
111	HOXB2	0.43723	Homeobox	0	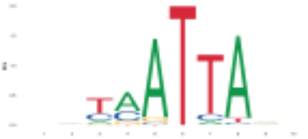
112	SPI1	0.44034	Ets	0	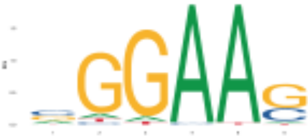
113	CREB3	0.44888	bZIP_1	0	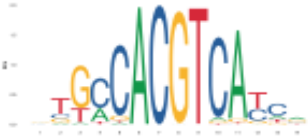
114	SOX8	0.44961	HMG_box	1	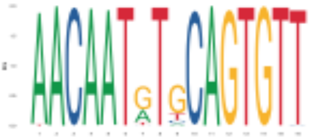
115	ID4	0.45057	HLH	0	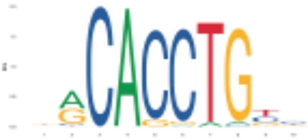
116	HOXB13	0.45087	Homeobox	0	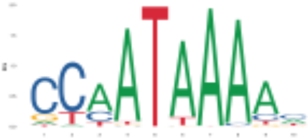
117	HOXC13	0.45087	Homeobox	0	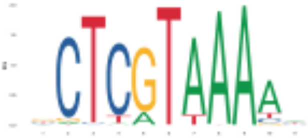
118	TBX15	0.45095	T−box	0	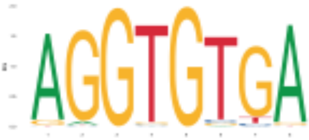
119	NFE2L2	0.45131	bZIP_1	0	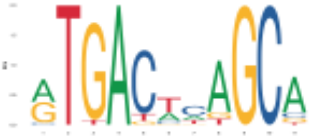
120	KLF14	0.45268	zf−C2H2	0	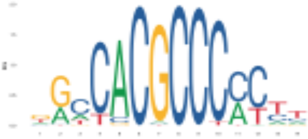

No.	TF	MPI	DBD family	Non redundant	Logo
121	XBP1	0.45558	bZIP_1	1	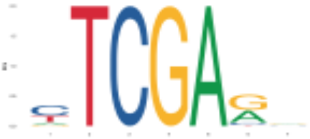
122	RELA	0.46232	RHD	1	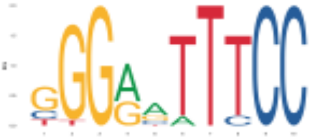
123	NRL	0.46316	bZIP_1	1	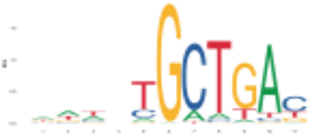
124	HNF1A	0.48833	Homeobox	1	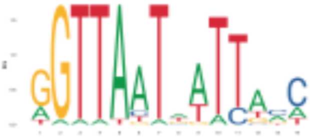
125	HNF1B	0.48833	Homeobox	0	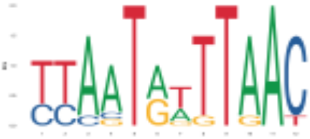
126	EOMES	0.49132	T−box	0	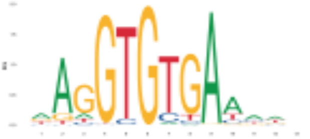
127	TBR1	0.49132	T−box	0	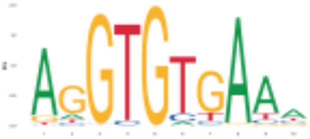
128	MAX	0.50234	HLH,bZIP_1	0	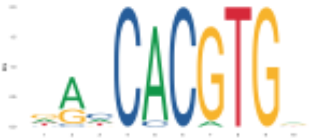
129	KLF16	0.50262	zf−C2H2	0	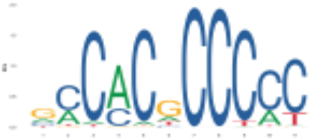
130	PPARG	0.5054	zf−C4	0	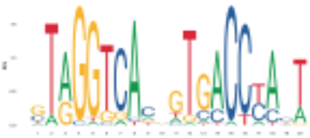

No.	TF	MPI	DBD family	Non redundant	Logo
131	ESR1	0.50667	zf−C4	0	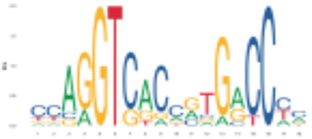
132	ESR2	0.50667	zf−C4	1	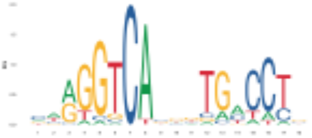
133	MAFF	0.50766	bZIP_1	0	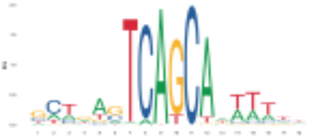
134	MAFG	0.51271	bZIP_1	0	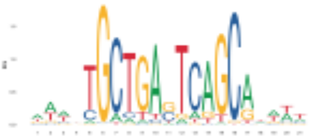
135	MAFK	0.51271	bZIP_1	0	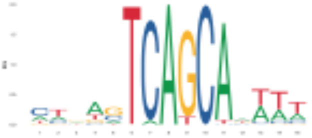
136	VSX1	0.51393	Homeobox	0	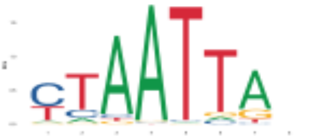
137	TFAP2C	0.51723	TF_AP−2	0	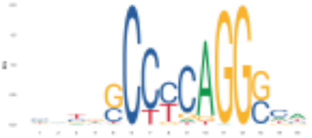
138	GSC2	0.51883	Homeobox	0	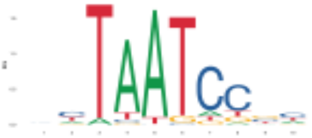
139	CEBPA	0.52439	bZIP_1	0	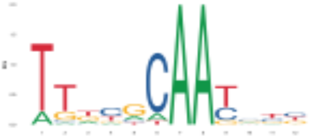
140	CEBPB	0.52439	bZIP_1	0	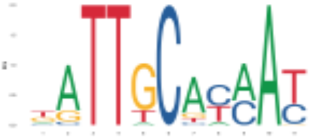

No.	TF	MPI	DBD family	Non redundant	Logo
141	CEBPE	0.52439	bZIP_1	0	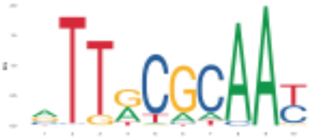
142	PROP1	0.52714	Homeobox	0	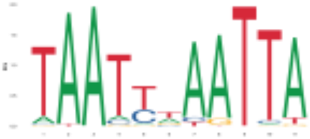
143	OLIG1	0.53773	HLH	0	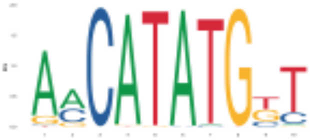
144	MNT	0.53803	HLH	0	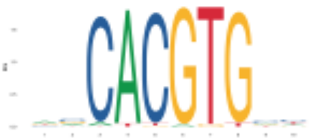
145	JDP2	0.54864	bZIP_1	0	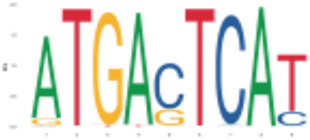
146	RUNX3	0.54876	Runt	0	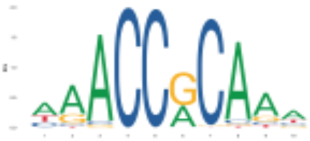
147	RUNX2	0.54945	Runt	1	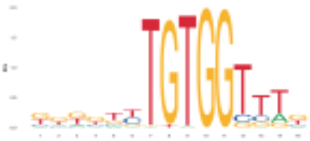
148	ZNF143	0.55096	zf−C2H2	1	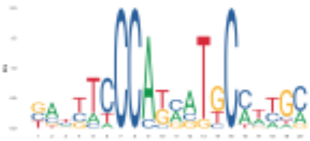
149	LEF1	0.55369	HMG_box	0	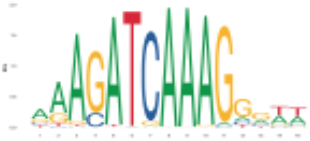
150	RARA	0.56479	zf−C4	0	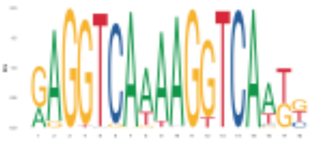

No.	TF	MPI	DBD family	Non redundant	Logo
151	TCF7L2	0.56994	HMG_box	1	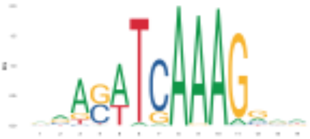
152	PKNOX1	0.57124	Homeobox	0	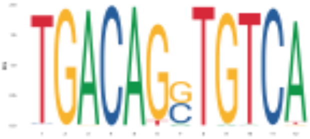
153	USF2	0.57978	HLH	0	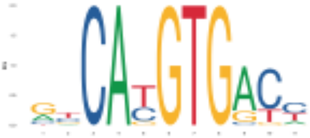
154	HOXC11	0.57993	Homeobox	0	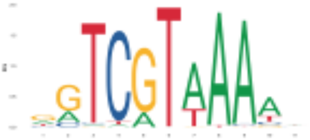
155	ETV6	0.59132	Ets	0	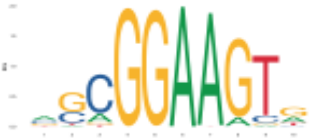
156	HOXD11	0.59211	Homeobox	1	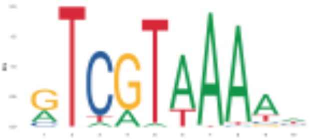
157	USF1	0.60326	HLH	0	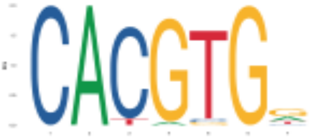
158	POU6F1	0.60664	Homeobox	0	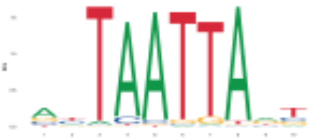
159	GLIS3	0.60724	zf−C2H2	0	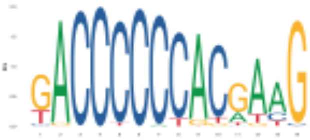
160	E2F4	0.60962	E2F_TDP	1	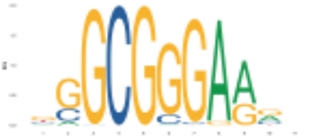

No.	TF	MPI	DBD family	Non redundant	Logo
161	SOX21	0.61944	HMG_box	1	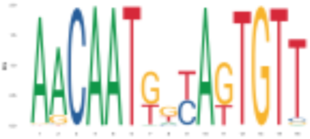
162	CLOCK	0.62712	HLH	0	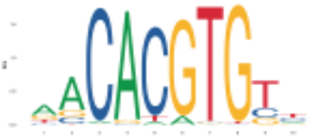
163	SCRT2	0.63575	zf−C2H2	0	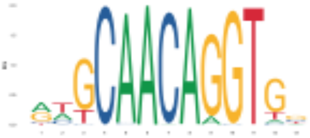
164	CEBPG	0.63686	bZIP_1	0	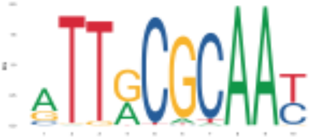
165	FOSL1	0.63773	bZIP_1	0	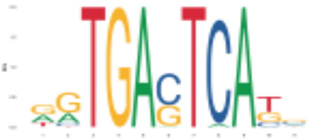
166	NFIL3	0.63845	bZIP_1	0	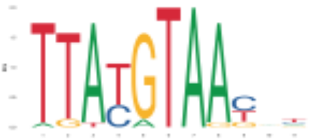
167	SNAI2	0.65255	zf−C2H2	0	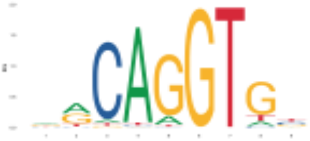
168	GSC	0.65388	Homeobox	0	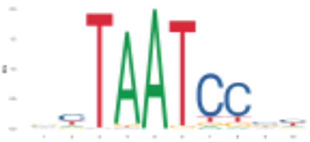
169	ZIC1	0.65976	zf−C2H2	1	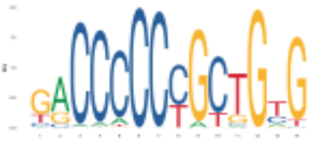
170	ZIC3	0.65976	zf−C2H2	0	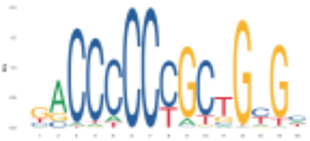

No.	TF	MPI	DBD family	Non redundant	Logo
171	ZIC4	0.65976	zf−C2H2	0	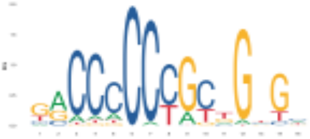
172	JUN	0.668	bZIP_1	0	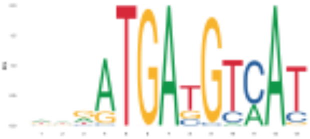
173	JUNB	0.668	bZIP_1	0	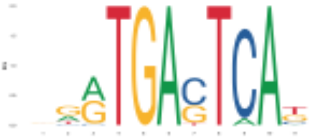
174	JUND	0.668	bZIP_1	0	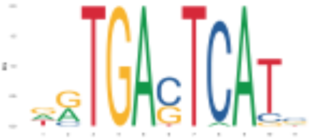
175	FOSL2	0.6691	bZIP_1	0	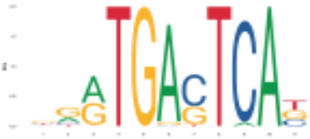
176	CREB3L1	0.67095	bZIP_1	0	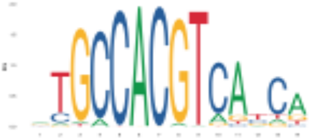
177	TGIF1	0.68104	Homeobox	0	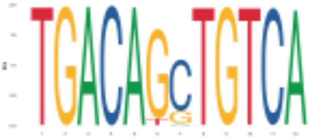
178	TGIF2	0.68104	Homeobox	0	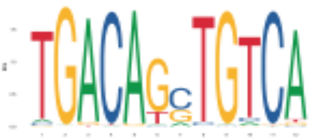
179	ESRRA	0.68434	zf−C4	1	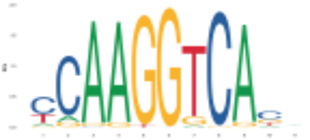
180	ESRRB	0.68434	zf−C4	0	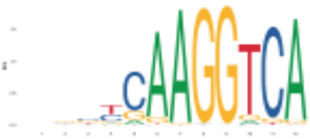

No.	TF	MPI	DBD family	Non redundant	Logo
181	MEOX1	0.68871	Homeobox	0	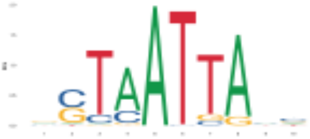
182	MEOX2	0.68871	Homeobox	0	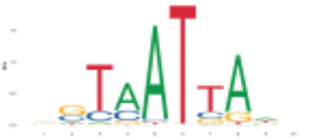
183	FOXO4	0.68956	Fork_head	0	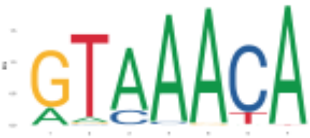
184	GCM1	0.6906	GCM	0	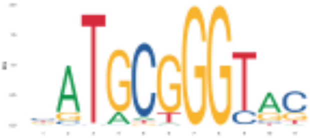
185	FOS	0.69327	bZIP_1	1	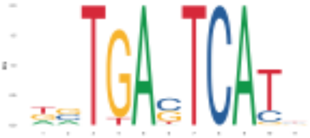
186	SMAD3	0.6979	MH1	1	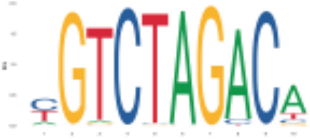
187	GBX1	0.70246	Homeobox	0	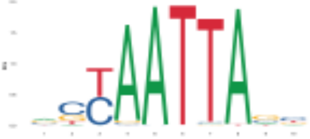
188	GBX2	0.70246	Homeobox	0	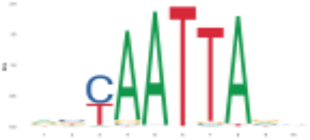
189	MLX	0.70249	HLH	0	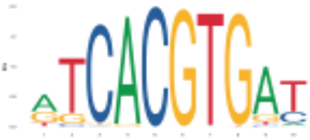
190	ELF5	0.70438	Ets	0	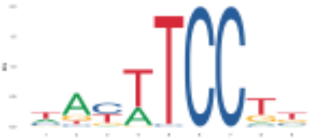

No.	TF	MPI	DBD family	Non redundant	Logo
191	LBX1	0.70898	Homeobox	0	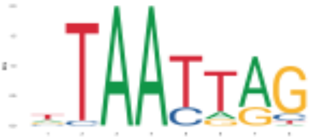
192	LBX2	0.70898	Homeobox	0	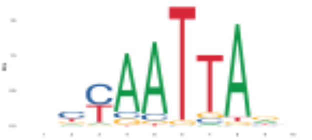
193	HOXC10	0.7116	Homeobox	0	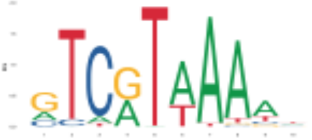
194	TBX1	0.71433	T−box	0	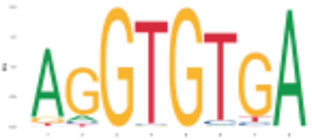
195	T	0.71575	T−box	0	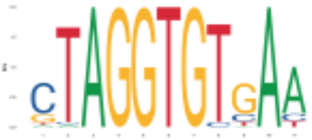
196	TBX19	0.71575	T−box	0	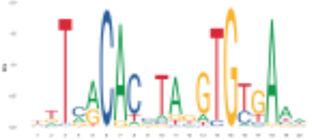
197	BARX1	0.71625	Homeobox	0	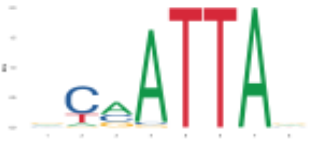
198	SCRT1	0.71771	zf−C2H2	0	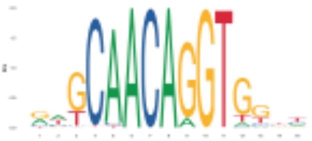
199	CDX1	0.71911	Homeobox	1	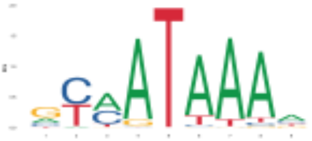
200	CDX2	0.71911	Homeobox	0	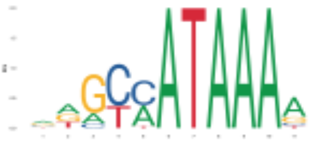

No.	TF	MPI	DBD family	Non redundant	Logo
201	EHF	0.73464	Ets	0	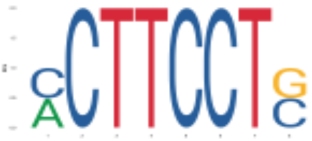
202	PAX3	0.73993	Homeobox,PAX	1	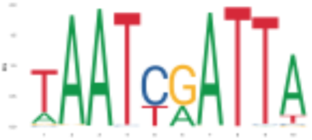
203	PAX7	0.73993	Homeobox,PAX	0	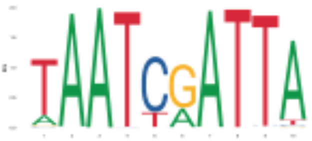
204	SRF	0.74233	SRF−TF	1	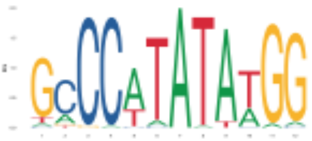
205	MNX1	0.74244	Homeobox	0	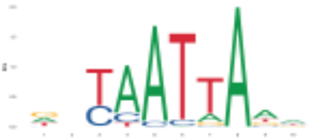
206	PAX6	0.74445	Homeobox,PAX	0	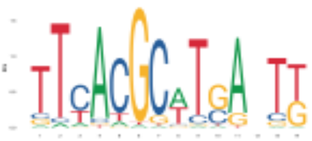
207	HNF4A	0.74487	zf−C4	0	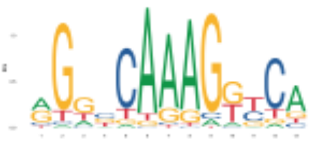
208	SREBF2	0.7452	HLH	0	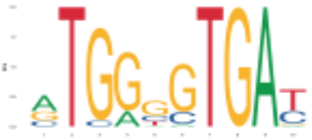
209	ELF3	0.74599	Ets	0	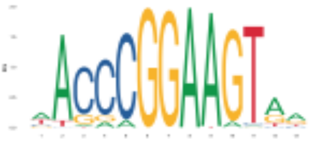
210	DBP	0.74611	bZIP_1	0	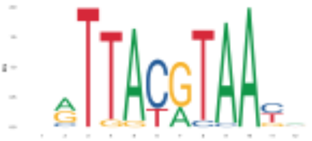

No.	TF	MPI	DBD family	Non redundant	Logo
211	TEF	0.74611	bZIP_1	0	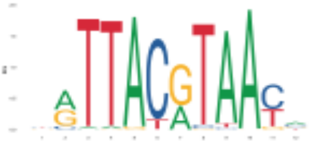
212	GLI2	0.74655	zf−C2H2	1	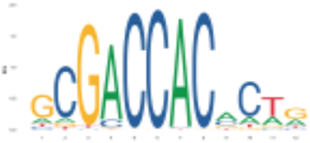
213	TFAP2A	0.75323	TF_AP−2	0	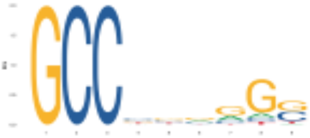
214	FOXO3	0.75649	Fork_head	0	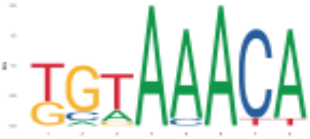
215	TFAP2B	0.75851	TF_AP−2	0	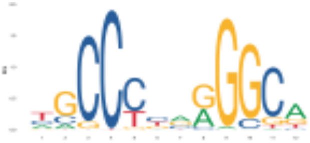
216	EN1	0.76182	Homeobox	0	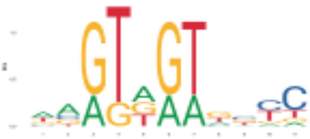
217	GSX1	0.76458	Homeobox	0	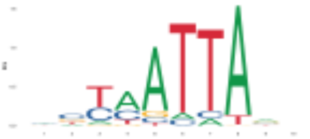
218	GSX2	0.76458	Homeobox	0	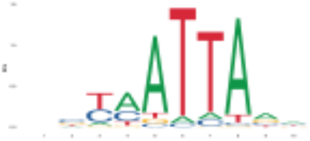
219	MSC	0.76681	HLH	0	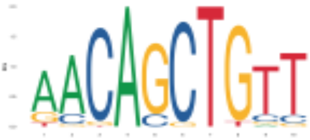
220	NR2F1	0.76722	zf−C4	0	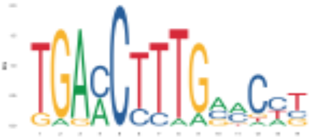

No.	TF	MPI	DBD family	Non redundant	Logo
221	NKX3−1	0.76778	Homeobox	0	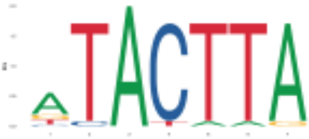
222	NKX3−2	0.76778	Homeobox	0	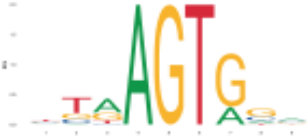
223	TBX4	0.77263	T−box	0	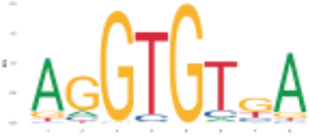
224	TBX5	0.77263	T−box	0	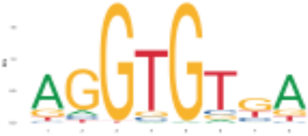
225	NHLH1	0.77584	HLH	0	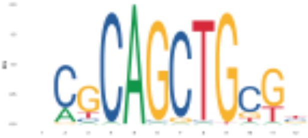
226	PKNOX2	0.77673	Homeobox	0	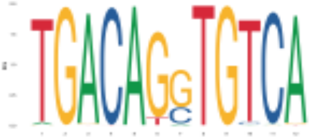
227	GCM2	0.77707	GCM	1	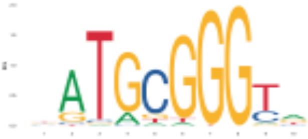
228	POU3F1	0.78229	Homeobox,Pou	0	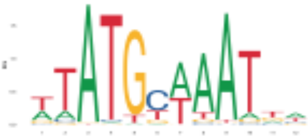
229	POU2F2	0.78415	Homeobox,Pou	0	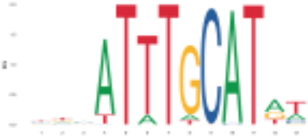
230	YY2	0.78603	zf−C2H2	1	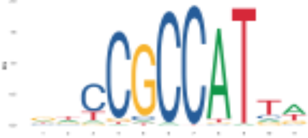

No.	TF	MPI	DBD family	Non redundant	Logo
231	EMX1	0.78769	Homeobox	0	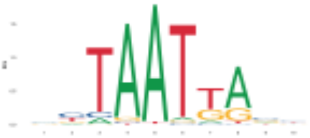
232	TFEC	0.78774	HLH	0	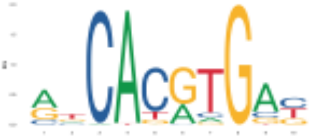
233	SPDEF	0.78812	Ets	0	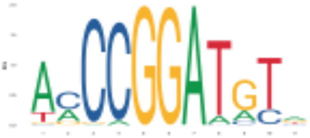
234	BSX	0.78889	Homeobox	0	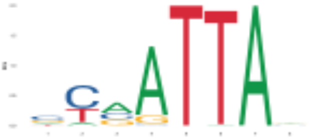
235	ONECUT3	0.79209	CUT,Homeobox	0	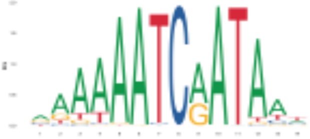
236	POU3F2	0.79323	Homeobox,Pou	0	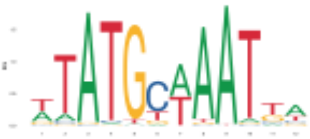
237	POU3F3	0.79323	Homeobox,Pou	0	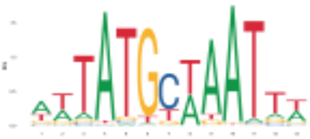
238	YY1	0.79343	zf−C2H2	1	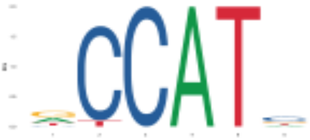
239	ONECUT1	0.79666	CUT,Homeobox	0	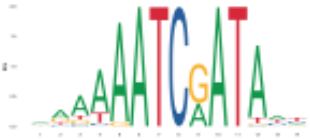
240	KLF13	0.79697	zf−C2H2	0	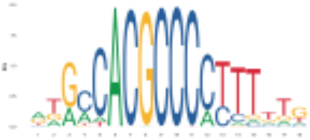

No.	TF	MPI	DBD family	Non redundant	Logo
241	POU3F4	0.7983	Homeobox,Pou	0	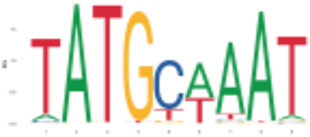
242	RORA	0.79998	zf−C4	0	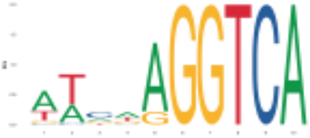
243	EMX2	0.80062	Homeobox	0	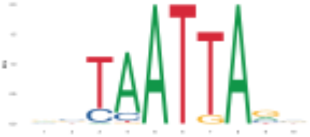
244	HLF	0.80195	bZIP_1	1	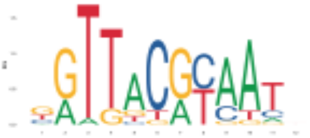
245	BARHL2	0.80406	Homeobox	1	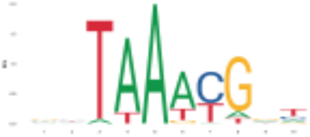
246	EN2	0.80506	Homeobox	0	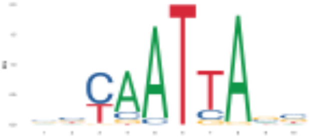
247	FOXO6	0.80617	Fork_head	0	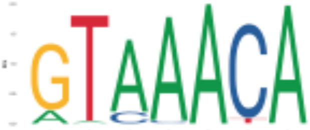
248	HNF4G	0.80663	zf−C4	0	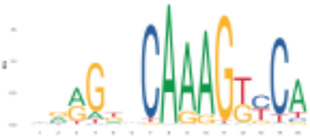
249	BHLHE22	0.81131	HLH	0	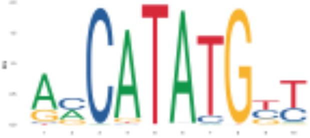
250	BHLHE23	0.81131	HLH	0	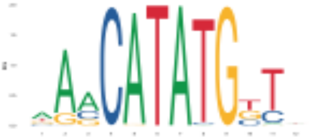

No.	TF	MPI	DBD family	Non redundant	Logo
251	OLIG2	0.81131	HLH	0	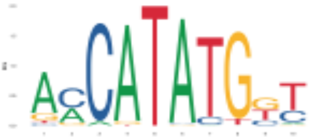
252	OLIG3	0.81131	HLH	0	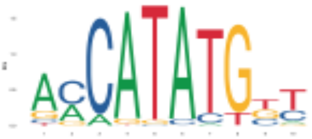
253	ONECUT2	0.81294	CUT,Homeobox	1	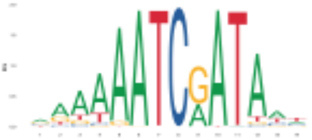
254	MEIS1	0.81363	Homeobox	1	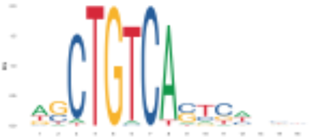
255	MEIS2	0.81363	Homeobox	0	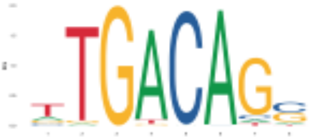
256	SREBF1	0.82229	HLH	0	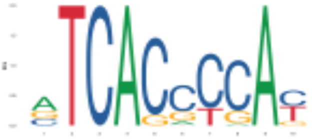
257	RXRB	0.82646	zf−C4	0	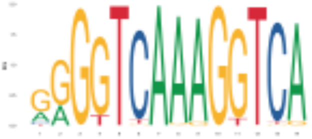
258	RXRG	0.82646	zf−C4	0	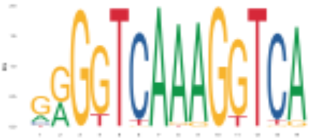
259	EVX1	0.83565	Homeobox	1	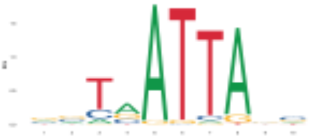
260	EVX2	0.83565	Homeobox	0	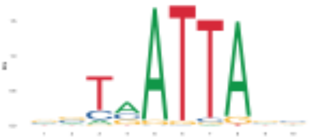

No.	TF	MPI	DBD family	Non redundant	Logo
261	LMX1A	0.83598	Homeobox	0	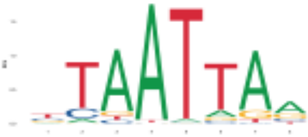
262	LMX1B	0.83598	Homeobox	0	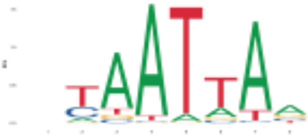
263	HOXB3	0.83713	Homeobox	0	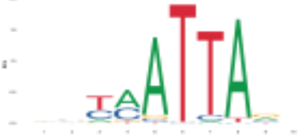
264	PITX3	0.8393	Homeobox	0	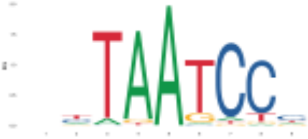
265	POU2F1	0.83999	Homeobox,Pou	1	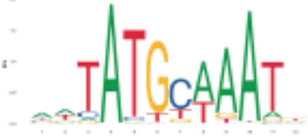
266	POU4F1	0.84085	Homeobox,Pou	1	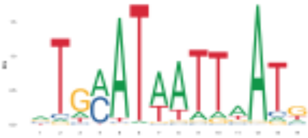
267	POU4F2	0.84085	Homeobox,Pou	0	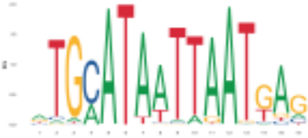
268	POU4F3	0.84085	Homeobox,Pou	0	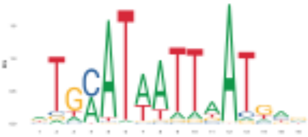
269	NR2C2	0.84715	zf−C4	1	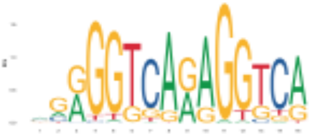
270	PAX1	0.85221	PAX	0	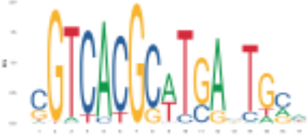

No.	TF	MPI	DBD family	Non redundant	Logo
271	PAX9	0.85221	PAX	0	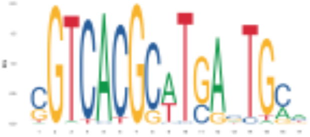
272	NEUROG2	0.85281	HLH	0	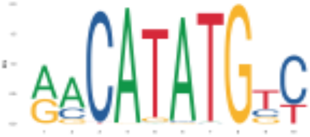
273	SHOX	0.85319	Homeobox	0	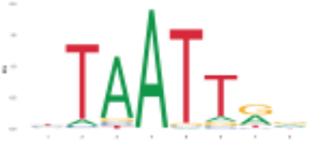
274	SP2	0.85419	zf−C2H2	0	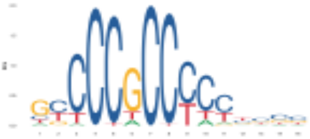
275	NR4A2	0.85697	zf−C4	1	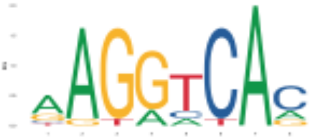
276	TFAP4	0.85932	HLH	0	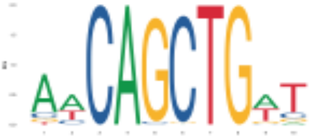
277	EGR1	0.85942	zf−C2H2	0	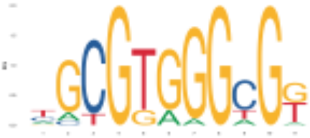
278	EGR2	0.85942	zf−C2H2	1	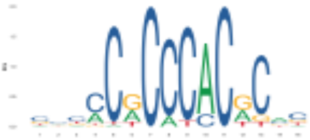
279	EGR3	0.85942	zf−C2H2	0	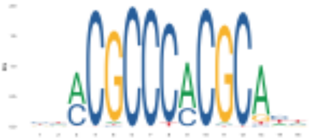
280	EGR4	0.85942	zf−C2H2	0	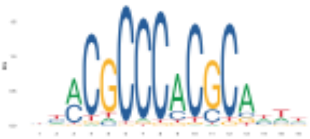

No.	TF	MPI	DBD family	Non redundant	Logo
281	TFE3	0.86467	HLH	1	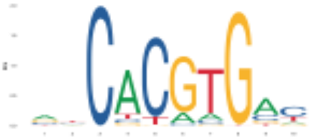
282	TFEB	0.86467	HLH	0	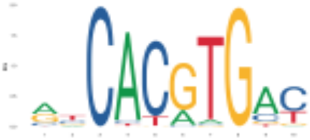
283	TBX2	0.86473	T−box	0	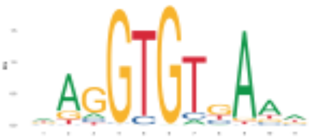
284	NFIA	0.87078	MH1	1	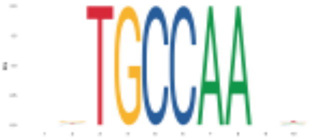
285	NFIC	0.87078	MH1	0	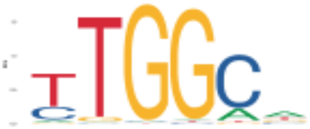
286	NFIX	0.87078	MH1	0	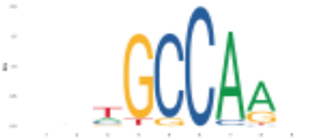
287	NKX6−1	0.87119	Homeobox	0	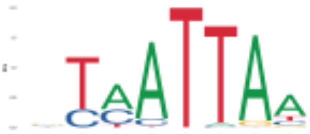
288	NKX6−2	0.87119	Homeobox	0	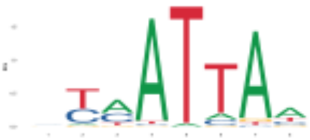
289	NFYA	0.87183	CBFB_NFYA	1	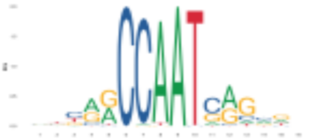
290	DLX6	0.87234	Homeobox	0	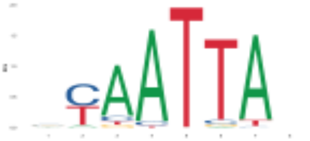

No.	TF	MPI	DBD family	Non redundant	Logo
291	ISX	0.87757	Homeobox	0	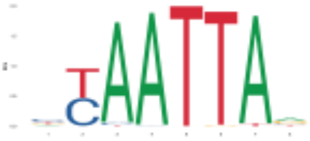
292	TBX20	0.88389	T−box	1	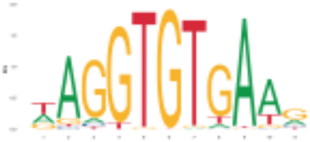
293	FOXP1	0.88735	Fork_head	0	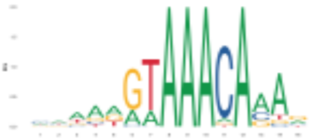
294	FOXP2	0.88735	Fork_head	0	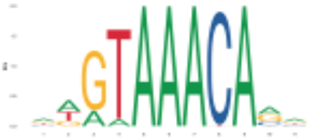
295	MYF6	0.88929	HLH	1	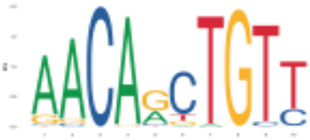
296	GATA5	0.89162	GATA	0	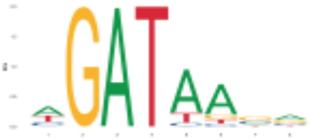
297	PAX5	0.89534	PAX	1	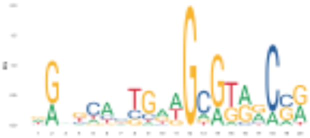
298	NEUROD2	0.89605	HLH	1	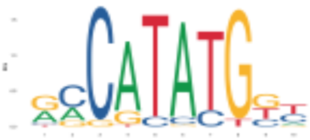
299	LHX9	0.9016	Homeobox	0	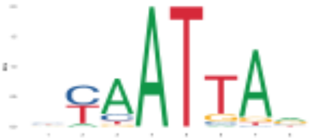
300	PBX1	0.9016	Homeobox	1	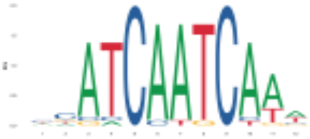

No.	TF	MPI	DBD family	Non redundant	Logo
301	PDX1	0.9016	Homeobox	0	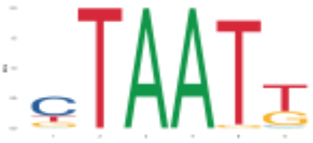
302	NKX2−8	0.9029	Homeobox	0	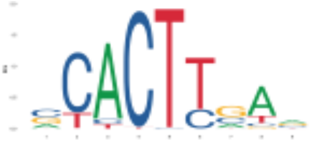
303	HEY1	0.90381	HLH	1	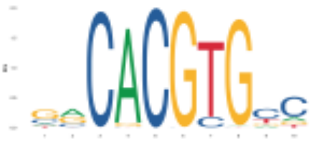
304	HEY2	0.90381	HLH	0	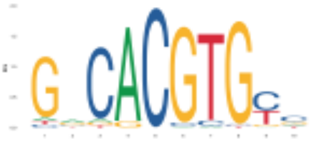
305	SP8	0.91145	zf−C2H2	0	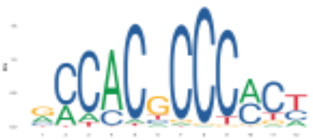
306	GATA3	0.91309	GATA	0	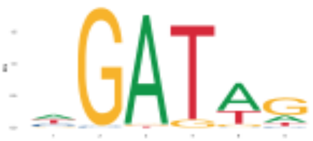
307	SP1	0.91726	zf−C2H2	0	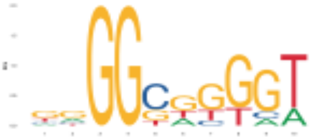
308	SP3	0.91726	zf−C2H2	0	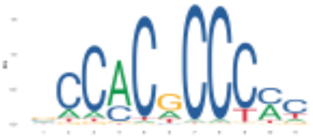
309	SP4	0.91726	zf−C2H2	0	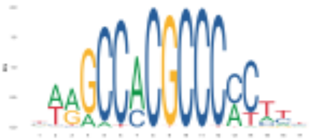
310	ELF1	0.91778	Ets	0	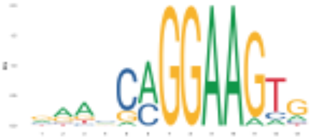

No.	TF	MPI	DBD family	Non redundant	Logo
311	ELF4	0.91778	Ets	0	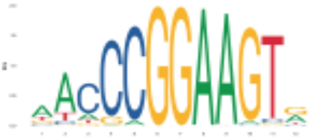
312	MSX1	0.91889	Homeobox	0	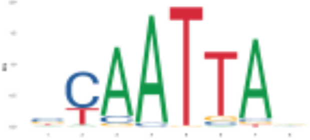
313	MSX2	0.91889	Homeobox	0	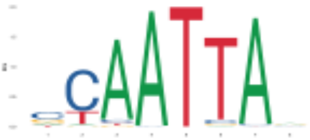
314	HOXA2	0.92307	Homeobox	0	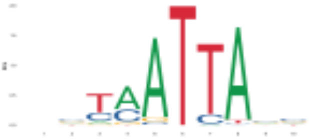
315	HOXA5	0.92307	Homeobox	1	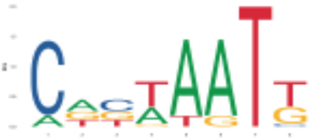
316	LHX2	0.92307	Homeobox	0	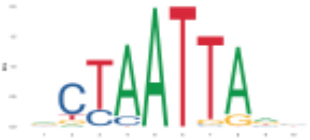
317	MEF2A	0.92307	SRF−TF	1	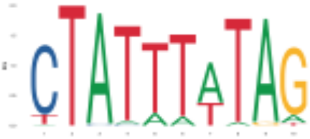
318	MEF2B	0.92307	SRF−TF	0	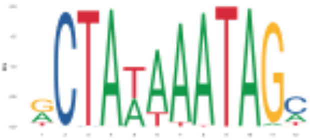
319	MEF2C	0.92307	SRF−TF	0	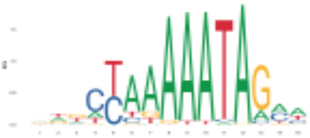
320	MEF2D	0.92307	SRF−TF	0	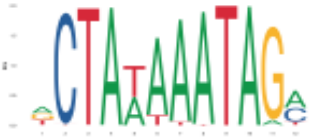

No.	TF	MPI	DBD family	Non redundant	Logo
321	TCF3	0.92314	HLH	1	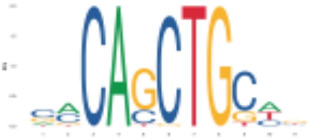
322	TCF4	0.92314	HLH	0	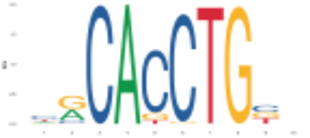
323	OTX1	0.93177	Homeobox	0	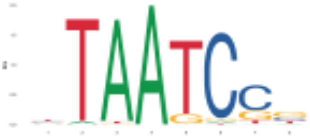
324	KLF5	0.9353	zf−C2H2	1	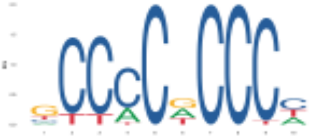
325	OTX2	0.95017	Homeobox	1	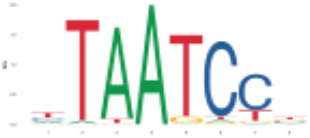
326	CREB1	0.95468	bZIP_1	1	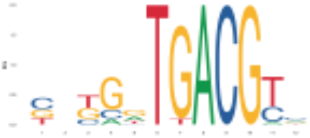
327	ISL2	0.95987	Homeobox	1	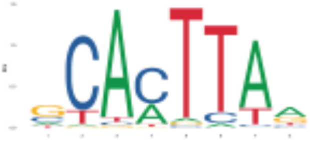
328	RAX2	0.96699	Homeobox	0	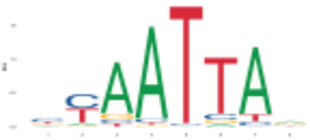
329	UNCX	0.97714	Homeobox	0	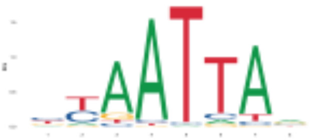
330	RAX	0.97853	Homeobox	0	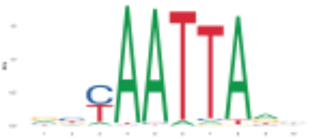

No.	TF	MPI	DBD family	Non redundant	Logo
331	VSX2	0.97853	Homeobox	0	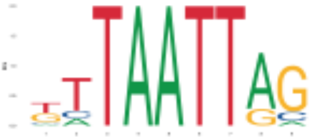
332	NKX2−3	0.98488	Homeobox	1	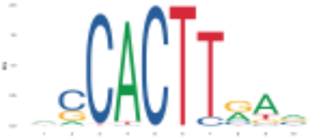
333	GATA2	0.99002	GATA	1	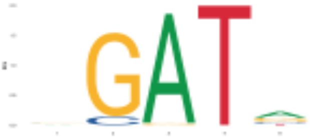
334	ELK1	1	Ets	1	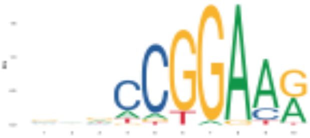
335	ELK3	1	Ets	0	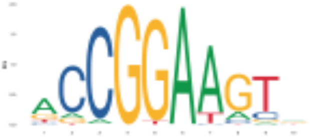
336	ELK4	1	Ets	0	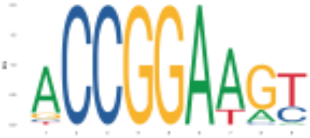
337	ERF	1	Ets	0	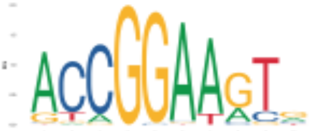
338	ERG	1	Ets	0	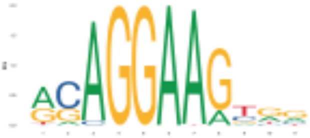
339	ETS1	1	Ets	0	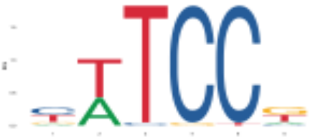
340	ETV1	1	Ets	0	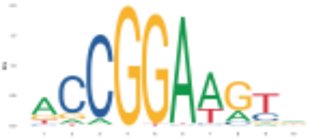

No.	TF	MPI	DBD family	Non redundant	Logo
341	ETV2	1	Ets	0	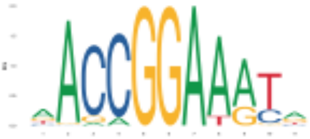
342	ETV3	1	Ets	0	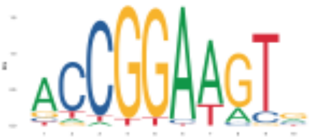
343	ETV4	1	Ets	0	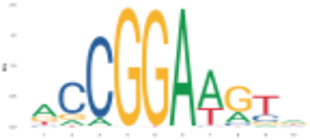
344	ETV5	1	Ets	0	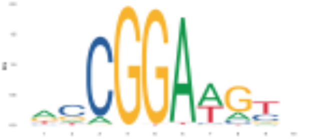
345	FEV	1	Ets	0	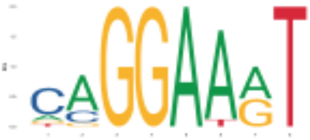
346	FLI1	1	Ets	0	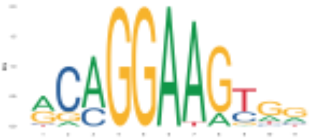
347	GABPA	1	Ets	0	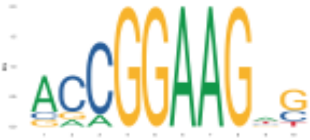
348	FOXA1	1	Fork_head	1	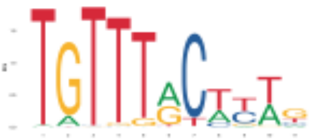
349	FOXB1	1	Fork_head	0	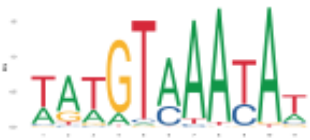
350	FOXC1	1	Fork_head	0	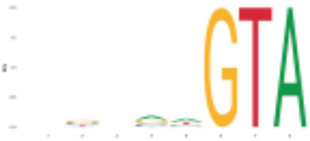

No.	TF	MPI	DBD family	Non redundant	Logo
351	FOXC2	1	Fork_head	0	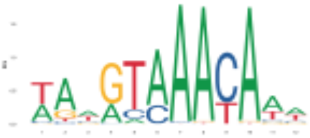
352	FOXD1	1	Fork_head	0	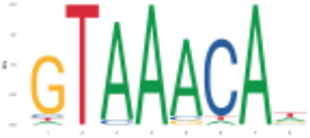
353	FOXD2	1	Fork_head	0	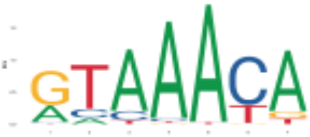
354	FOXF2	1	Fork_head	1	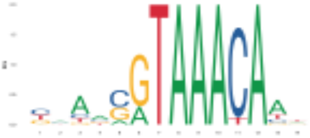
355	FOXG1	1	Fork_head	0	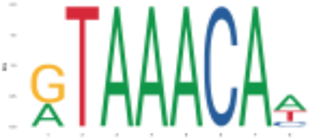
356	FOXI1	1	Fork_head	0	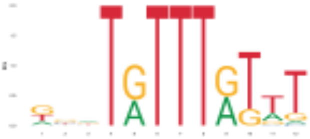
357	FOXL1	1	Fork_head	0	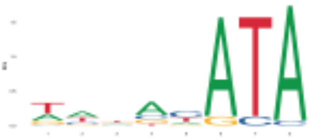
358	ALX3	1	Homeobox	1	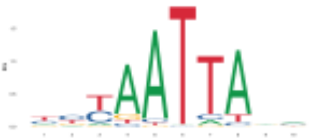
359	PHOX2A	1	Homeobox	0	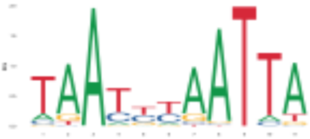
360	PRRX1	1	Homeobox	0	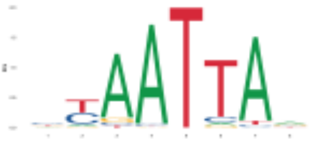

No.	TF	MPI	DBD family	Non redundant	Logo
361	RFX2	1	RFX_DNA_binding	1	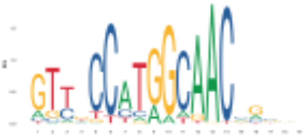
362	RFX3	1	RFX_DNA_binding	0	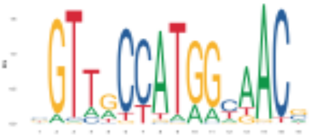
363	RFX4	1	RFX_DNA_binding	0	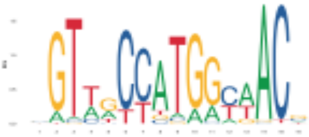
364	RFX5	1	RFX_DNA_binding	0	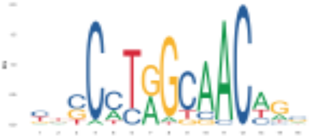
