# Supplementary material for: Borders of Cis-Regulatory DNA Sequences Preferentially Harbor the Divergent Transcription Factor Binding Motifs in the Human Genome
Source: Front Genet. 2018 Nov 22;9:571. doi: 10.3389/fgene.2018.00571 (PMC6261980; doi:10.3389/fgene.2018.00571)
Supplement: Supplementary file 5 [file Data_Sheet_3.PDF]

Supplementary Table S4. The significant values for the differences of mean MPI scores between DHS-center and DHS-edge overlapped with TF-ChIP among different *cis* -regulatory regions.

DHSs in the cis-regulatory regions	*P* -values (Wilcoxon rank sum test)
Promoter of protein coding genes	3.76E-11
Promoter of pseudogenes	2.07E-04
Promoter of non-coding genes	6.85E-07
Enhancers (FANTOM5, Andersson et al. 2014)	1.01E-10
Enhancers (VISTA, Visel et al., 2007)	1.01E-02
Others	9.22E-33
